# Thyroid function and mood disorders: a Mendelian Randomization study

**DOI:** 10.1089/thy.2020.0884

**Published:** 2021-05-26

**Authors:** Aleksander Kuś, Alisa D. Kjaergaard, Eirini Marouli, M. Fabiola Del Greco, Rosalie B.T.M. Sterenborg, Layal Chaker, Robin P. Peeters, Tomasz Bednarczuk, Bjørn O. Åsvold, Stephen Burgess, Panos Deloukas, Alexander Teumer, Christina Ellervik, Marco Medici

**Affiliations:** 1 Academic Center for Thyroid Diseases, Department of Internal Medicine, Erasmus Medical Center, Dr. Molewaterplein 40, 3015 CE Rotterdam, The Netherlands; 2 Department of Epidemiology, Erasmus Medical Center, Dr. Molewaterplein 50, 3015 GE Rotterdam, The Netherlands; 3 Department of Internal Medicine and Endocrinology, Medical University of Warsaw, Banacha 1a, 02-097 Warsaw, Poland; 4 Steno Diabetes Center Aarhus, Aarhus University Hospital, Hedeager 3, 8000 Aarhus, Denmark; 5 William Harvey Research Institute, Barts and The London School of Medicine and Dentistry, Queen Mary University of London, EC1M 6BQ London, UK; 6 Centre for Genomic Health, Life Sciences, Queen Mary University of London, EC1M 6BQ London, UK; 7 Institute for Biomedicine, Eurac Research, Affiliated Institute of the University of Lubeck, Via Galvani 31, 39100 Bolzano, Italy; 8 Department of Internal Medicine, Radboud University Medical Center, Geert Grooteplein Zuid 10, 6525 GA Nijmegen, The Netherlands; 9 K.G. Jebsen Center for Genetic Epidemiology, NTNU, Norwegian University of Science and Technology, Post box 8905, 7491 Trondheim, Norway; 10 Department of Endocrinology, St. Olavs Hospital, Trondheim University Hospital, Prinsesse Kristinas gate 3, 7030 Trondheim, Norway; 11 MRC Biostatistics Unit, Cambridge Institute of Public Health, University of Cambridge, Forvie Site, Robinson Way, Cambridge, CB2 0SR, UK; 12 Cardiovascular Epidemiology Unit, Department of Public Health and Primary Care, University of Cambridge, 2 Worts Causeway, Cambridge CB1 8RN, UK; 13 Princess Al-Jawhara Al-Brahim Centre of Excellence in Research of Hereditary Disorders (PACER-HD), King Abdulaziz University, Jeddah, Saudi Arabia; 14 Institute for Community Medicine, University Medicine Greifswald, W.-Rathenau-Str. 48, 17475 Greifswald, Germany; 15 DZHK (German Center for Cardiovascular Research), partner site Greifswald, Fleischmannstr. 8, 17475 Greifswald, Germany; 16 Department of Laboratory Medicine, Boston Children's Hospital, 300 Longwood Avenue, Boston, 02115 MA, USA; 17 Harvard Medical School, 25 Shattuck St, Boston, MA 02115, USA; 18 Department of Clinical Medicine, Faculty of Health and Medical Sciences, University of Copenhagen, Nørre Alle 41, 2200 Copenhagen, Denmark

**Keywords:** Mendelian randomization study, normal-range thyroid function, thyroid, depression, bipolar disorder, mood disorders

## Abstract

**Background:**

Observational studies suggest that even minor variations in thyroid function are associated with the risk of mood disorders, including major depressive disorder (MDD) and bipolar disorder (BD). However, it is unknown whether these associations are causal or not. We used a Mendelian Randomization (MR) approach to investigate causal effects of minor variations in TSH and FT4 levels on MDD and BD risk.

**Methods:**

We performed two-sample MR analyses using data from the largest publicly available genome-wide association studies on normal-range TSH (N=54,288) and FT4 (N=49,269) levels, MDD (170,756 cases, 329,443 controls) and BD (20,352 cases, 31,358 controls). Secondary MR analyses investigated the effects of TSH and FT4 levels on specific MDD and BD subtypes. Reverse MR was also performed to assess the effects of MDD and BD on TSH and FT4 levels.

**Results:**

There were no associations between genetically predicted TSH and FT4 levels and MDD risk, nor MDD subtypes and minor depressive symptoms. A one standard deviation increase in FT4 levels was nominally associated with an 11% decrease in the overall BD risk (OR=0.89, 95%CI=0.80-0.98, *P*=0.022) and a 13% decrease in the BD type 1 risk (OR=0.87, 95%CI=0.75-1.00, *P*=0.047). In the reverse direction, genetic predisposition to MDD and BD was not associated with TSH nor FT4 levels.

**Conclusions:**

Variations in normal-range TSH and FT4 levels have no effects on the risk of MDD and its subtypes, and neither on minor depressive symptoms. This indicates that depressive symptoms should not be attributed to minor variations in thyroid function. Borderline associations with BD and BD type 1 risks suggest that further clinical studies should investigate the effect of thyroid hormone treatment in BD.

## Introduction

Mood disorders, including major depressive disorder (MDD) and bipolar disorder (BD), have a global lifetime prevalence of up to 20% (1–3). It has been suggested that a complex interaction of social, psychological and biological factors is involved in the pathogenesis of MDD and BD (4), but a better understanding of the underlying mechanisms is still crucial for a further improvement in their prevention and treatment (5). Thyroid hormones are essential for neurocognitive development and function, and therefore the association between thyroid dysfunction and mood disorders has been extensively studied over the last decades (6, 7). Indeed, both hypo- and hyperthyroidism have been associated with MDD and/or BD in observational studies (8–11). More recently, even variation in normal-range thyroid function has been associated with MDD risk in large cohort studies (12–14). *Vice versa,* it has also been suggested that mood disorders affect the functioning of the hypothalamic-pituitary-thyroid (HPT) axis, as illustrated by a blunted thyrotropin (TSH) response to thyrotropin-releasing hormone (TRH) stimulation, and a decreased amplitude of the nocturnal TSH surge in patients with MDD (15–17). However, it is not clear whether the observed associations are causal, as observational studies are often prone to selection bias, residual confounding and reverse causality (18, 19). Several experimental studies reported beneficial effects of adjunct treatment with thyroid hormones in euthyroid patients with refractory mood disorders, including both MDD and BD (20–22). It has been suggested that thyroid hormones may accelerate the effect of antidepressant treatment as well as induce the response in those who do not respond to standard treatment (23, 24). However, the available randomized controlled trials (RCTs) on such treatments provided conflicting results, and none of them can be considered definitive due to their small sample sizes and/or short follow-up periods (25–28).

Taken together, the above studies suggest that thyroid function and mood disorders are closely related, but it is essential to clarify whether the observed associations are causal or not. Mendelian randomization (MR) is a commonly used approach that can provide the information on causality when RCTs are not feasible or lacking (29–34). This approach uses genetic variants as proxies to evaluate the causal effect of an exposure (*e.g.* thyroid function) on the outcome of interest (*e.g.* MDD or BD) (29). It draws from the fact that genetic variants segregate randomly from parents to offspring, which can be compared to randomization used in RCTs. As genetic variants can affect the outcome of interest but not the other way around, an association between the genetically predicted exposure and the tested outcome can provide evidence for causality (29).

In this novel study, we performed a two-sample MR to investigate the causal effects of variation in normal-range TSH and free thyroxine (FT4) levels on MDD and BD risk. In secondary analyses, we analyzed the effects of thyroid function on specific MDD and BD subtypes, while reverse MR analyses were performed to assess the effects of MDD and BD on TSH and FT4 levels.

## Materials and Methods

### Two-sample Mendelian randomization

**Figure 1:**
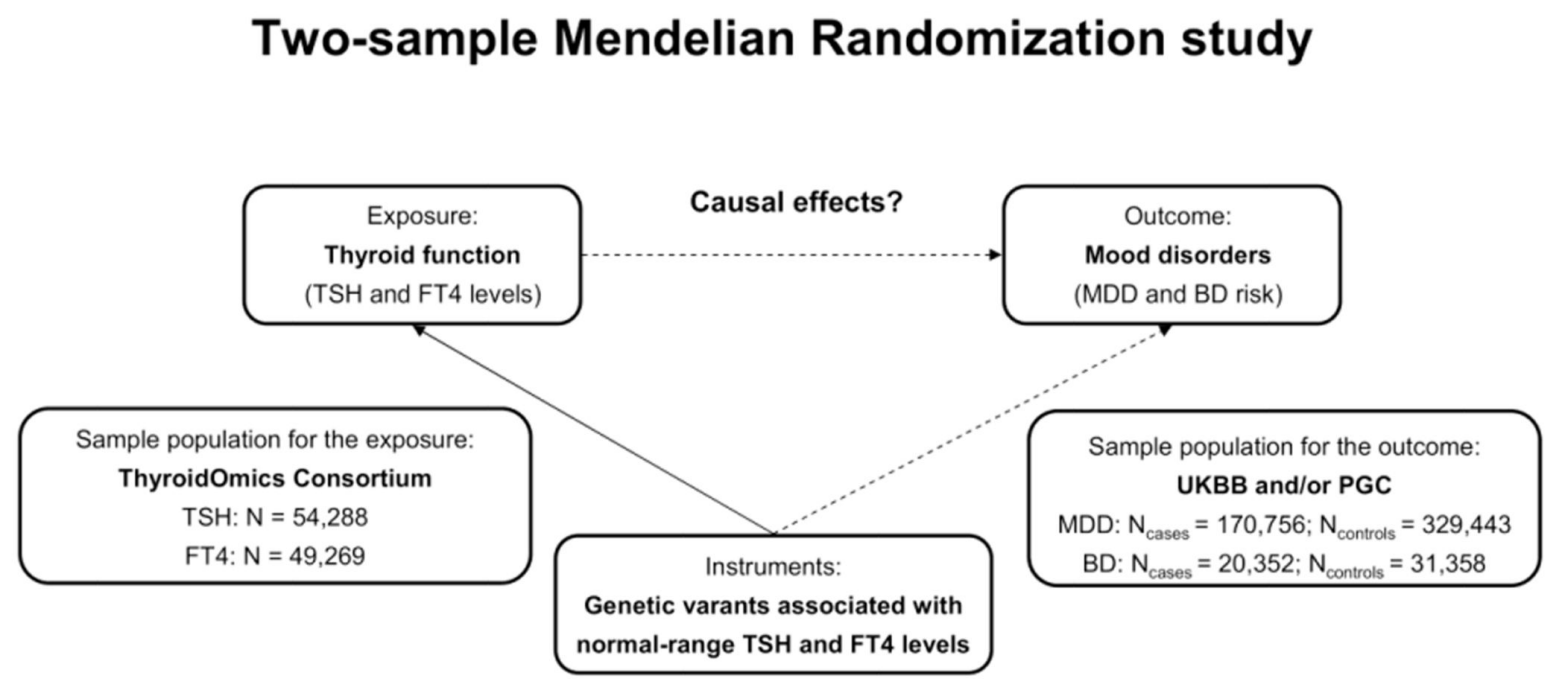
Schematic diagram illustrating the study design. Two-sample Mendelian Randomization (MR) approach based on the summary-level data from large-scale meta-analyses of the genome-wide association studies (GWAS) was used to investigate the causal effects of thyroid function on mood disorders. Genetic variants associated with normal-range TSH and FT4 levels (genetic instruments, represented by the solid line) and their corresponding effect estimates were established in the GWAS by the ThyroidOmics Consortium (35). Effect estimates on major depressive disorder (MDD) and bipolar disorder (BD) for these genetic variants were derived from the GWAS in the UK Biobank (UKBB) and/or the Psychiatric Genomics Consortium (PGC) (36–38). All datasets used in this study are publicly available at the ThyroidOmics Consortium and the PGC websites.

We performed two-sample MR analyses using the data from the most recent genome-wide association study (GWAS) on thyroid function (35) as exposures, and summary-level statistics from the largest publicly available GWAS meta-analyses on MDD and BD (36, 37) as outcomes (Figure 1). For secondary MR analyses we used the summary-level statistics from the GWAS on specific MDD and BD subtypes (detailed in the sections below and in Supplementary Tables 1 and 2; (37, 38)). For the reverse MR, the assignment of exposures and outcomes was switched for the analyses. No ethical approval was required as all data were extracted from publicly available summary statistics.

### Exposures (thyroid hormone levels) datasets

The exposures of interest were normal-range TSH and FT4 levels. Based on the results of a recent GWAS on thyroid function in the ThyroidOmics Consortium (35), we identified 61 and 31 independent (r^2^≤0.01 within windows of ±1 Mb for variants in the same locus) single nucleotide polymorphisms (SNPs) associated at a genome-wide significant level (*P*<5x10^-8^) with normal-range TSH and FT4 levels, respectively. Only individuals with TSH levels within their cohort-specific reference ranges were included in the GWAS on TSH (N=54,288) and FT4 (N=49,269) levels, and subjects using thyroid medications or after thyroid surgery were excluded from these GWAS, while no screening for mood disorders was performed among the participants in that study (35). We used the identified variants as instruments to investigate the causal relationship between normal-range thyroid function and the outcomes of interest. Two variants associated with TSH levels were *a priori* excluded from all analyses as they were highly pleiotropic (*ABO*-rs8176645, identified using the PhenoScanner v2 database available at: http://www.phenoscanner.medschl.cam.ac.uk/) or had the same effect allele associated (*P*<0.05) with both higher TSH levels and higher FT4 levels within the normal range (*BCAS3-* rs1157994). Detailed data on variants used as instruments are presented in Supplementary Tables 3 and 4.

### Outcomes (mood disorders) datasets

The primary outcomes of interest included MDD and BD. For the primary MDD analyses, we used the largest publicly available summary-level data derived from the GWAS meta-analysis by Howard *et al.* (36), including 33 cohorts of the Psychiatric Genomics Consortium (PGC) and UK Biobank (UKBB). This meta-analysis combined results of 500,199 individuals (170,756 cases and 329,443 controls). Case and control status for the broad depression phenotype in the UKBB cohort was defined by the primary or secondary diagnosis of a depressive mood disorder from linked hospital admission records or the participants' positive response to the questions “Have you ever seen a general practitioner for nerves, anxiety, tension or depression?” or “Have you ever seen a psychiatrist for nerves, anxiety, tension or depression?” in the online mental health questionnaire, with exclusions applied to participants who were identified with BD, schizophrenia, or personality disorder using self-declared data as well as those with prescriptions for antipsychotic medications (see Supplementary Table 2A) (36). The detailed inclusion/exclusion criteria for cases and controls for each of the PGC cohorts are available in the original manuscript by Wray *et al.* (39).

As the data on MDD subtypes were not available in the study by Howard *et al.* (36), we used the summary-level data derived from the GWAS on MDD in UKBB provided by Coleman *et al.* (38) for the secondary analyses with specific MDD subtypes. This study included 29,475 MDD cases and 63,482 controls identified based on the online mental health questionnaire. The definition of MDD in this cohort was based on DSM-5, as described in Supplementary Table 2B and in full elsewhere (40). Individuals meeting criteria for MDD were classified as “recurrent” if they reported multiple depressed periods across their lifetime (rMDD, N=17,451), and “single-episode” otherwise (sMDD, N=12,024; Supplementary Table 2B). Individuals reporting depressive symptoms but not meeting MDD case criteria were used as a “sub-threshold depression” subtype to examine the continuity of associations with MDD below clinical thresholds (subMDD, N=21,596).

For the MR analyses with BD and BD subtypes (including BD type 1, BD1; BD type 2, BD2; and schizoaffective BD, SABD), we used the summary-level data derived from the GWAS meta-analysis by Stahl *et al.* (37). This study meta-analyzed the results of 32 GWAS, totaling 20,352 cases and 31,358 controls of European descent (37). Cases were required to meet international consensus criteria (DSM-IV, ICD-9, or ICD-10) for a lifetime diagnosis of BD established using structured diagnostic instruments from assessments by trained interviewers, clinician-administered checklists, or medical record review. Controls in most samples were screened for the absence of lifetime psychiatric disorders. The detailed inclusion/exclusion criteria for cases and controls for each study included in the metaanalysis are available in the original manuscript by Stahl *et al.* (37).

All datasets used in this study are publicly available at the PGC website (https://www.med.unc.edu/pgc/download-results/). Data on the effect/other alleles, beta coefficients (β), standard errors (SE) and *P*-values for the variants associated with TSH and FT4 levels were extracted from each study for MR analyses and presented in Supplementary Tables 3 and 4.

### Statistical analyses

#### Main analyses

The main analyses included two-sample MR analyses performed using the inverse-variance weighted (IVW) method (41). This approach requires several assumptions as described in the Supplementary Materials and Methods. To control for false positive findings due to multiple testing, a conservative Bonferroni correction adjusted for the number of exposures and primary outcomes analyzed in the study was applied, and *P*-values less than 0.05/4=0.0125 were considered statistically significant in all analyses. A *P*-value less than 0.05 was considered as evidence for nominal significance. All analyses evaluate the causal effects of a one standard deviation (SD) increase in genetically predicted TSH or FT4 levels, approximately corresponding to a 1.0 mU/L and 2.2 pmol/L increase in TSH and FT4, respectively (42).

#### Sensitivity analyses and power calculations

We performed sensitivity analyses, reverse MR analyses and power calculations as described in detail in Supplementary Materials and Methods. In brief, **s**ensitivity analyses using various statistical MR methods (including MR Egger (43), weighted median (WM) (44), and MR-PRESSO (45)) were performed in order to account for potential pleiotropy in the associations between thyroid function and the outcomes of interest. Moreover, as autoimmunity in general has been associated with mood disorders (46–49), we repeated the analyses using as instruments two separate subsets of TSH associated variants (*i.e.* variants associated with autoimmunity thyroid disease (AITD) and variants not associated with AITD analyzed separately) in order to separate potential thyroid from autoimmunity mediated effects. Similarly, we also identified two separate subsets of FT4 associated variants, specifically including: (i) variants within the deiodinases loci (*i.e. DIO1* and *DIO2),* and (ii) other (non-deiodinase) genetic variants associated with FT4 levels in the GWAS by Teumer *et al.* (35). Furthermore, reverse MR analyses on TSH and FT4 levels and MDD and BD were performed to gain insight into the complex and potentially bidirectional associations between thyroid function and mood disorders, and power analysis was performed using a non-centrality parameter-based approach (50). The results of power calculations are provided in Supplementary Table 5.

## Results

**Figure 2:**
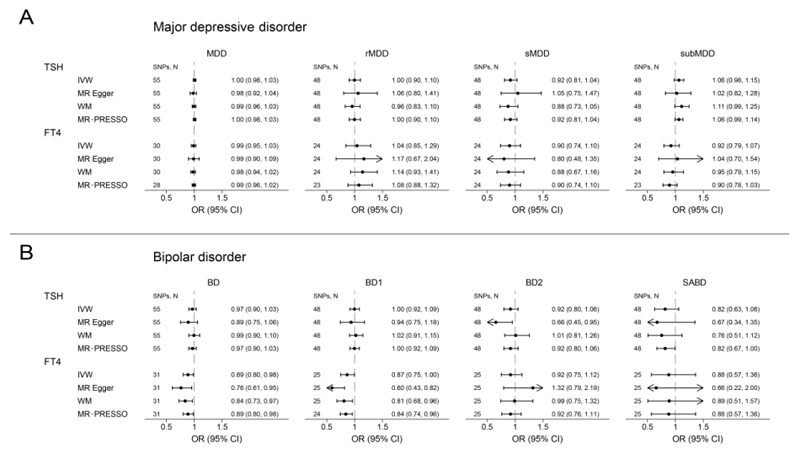
Causal effects of variation in normal-ranged thyrotropin (TSH) and free thyroxine (FT4) levels on major depressive disorder (A) and bipolar disorder (B) overall and specific subtypes risk. Presented odds ratios (OR) and confidence intervals (CI) correspond to the effects of a one standard deviation change in TSH and FT4 levels. The results of Mendelian Randomization (MR) analyses using various analysis methods (inverse variance weighted [IVW], MR-Egger, weighted median [WM], MR Pleiotropy RESidual Sum and Outlier [MR-PRESSO]) are presented for comparison. The number of Single Nucleotide Polymorphisms (SNPs) indicates the number of genetic variants used as instruments for MR analysis. rMDD - recurrent major depressive disorder; sMDD - single-episode major depressive disorder; subMDD - sub-threshold depression (*i.e.* individuals reporting depressive symptoms but not meeting formal major depressive disorder criteria); BD1 - bipolar disorder type 1; BD2 - bipolar disorder type 2; SABD - schizoaffective bipolar disorder.

**Figure 3:**
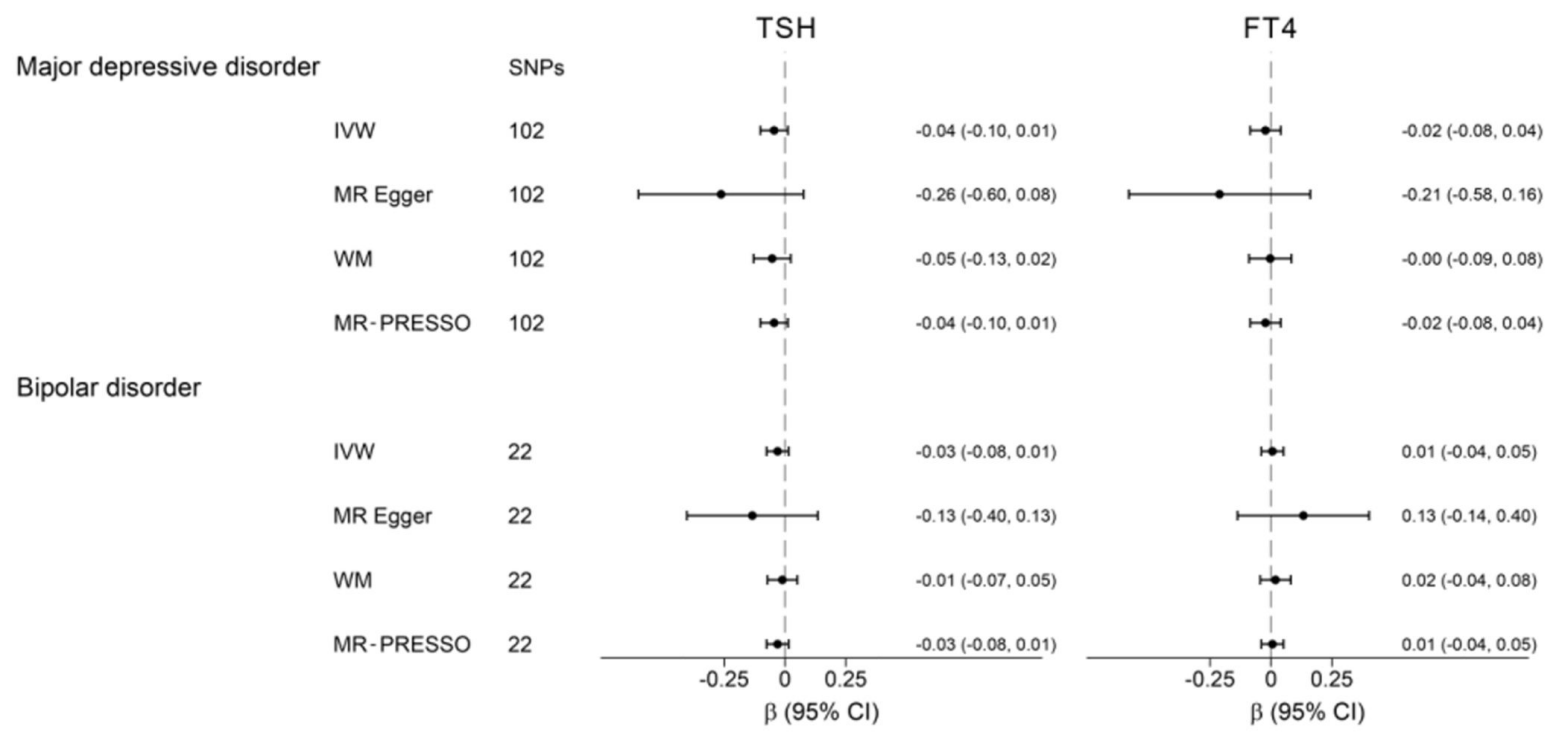
Causal effects of major depressive disorder and bipolar disorder on thyrotropin (TSH) and free thyroxine (FT4) levels. The results of Mendelian Randomization (MR) analyses using various analysis methods (inverse variance weighted [IVW], MR-Egger, weighted median [WM], MR Pleiotropy RESidual Sum and Outlier [MR-PRESSO]) are presented for comparison. The number of Single Nucleotide Polymorphisms (SNPs) indicates the number of genetic variants used as instruments for MR analysis.

The results of MR analyses investigating the associations between genetically predicted normal-range TSH and FT4 levels and MDD and BD risk are presented in Supplementary Tables 6-11 and summarized in Figures 2 and 3 and below.

### Major depressive disorder and subtypes

No associations were found between TSH and FT4 levels and MDD (OR=1.00, 95%CI=0.98-1.03, *P*= 0.68, and OR=0.99, 95%CI=0.95-1.03, *P*=0.56, respectively) or any of the MDD subtypes. Sensitivity analyses using the MR Egger and WM methods provided similar results (Figure 2). No evidence of directional pleiotropy was found based on the Egger intercept while exclusion of potentially pleiotropic variants identified using the MR-PRESSO method did not change the results (Supplementary Tables 7-8).

### Bipolar disorder and subtypes

A one SD increase in FT4 levels was nominally associated with a 11% decrease in the overall BD risk (OR=0.89, 95%CI=0.80-0.98, *P*=0.022) and a 13% decrease in the BD type 1 risk (OR=0.87, 95%CI=0.75-1.00, *P*=0.047; Figure 2), but these findings were not statistically significant after multiple testing correction. No associations were found between TSH levels and BD (OR=0.97, 95%CI=0.90-1.03, P=0.31) or any of the specific BD subtypes. Sensitivity analyses using the MR Egger, WM and MR-PRESSO methods provided similar results (Figure 2 and Supplementary Tables 6-8).

### Secondary analyses

#### MR analyses with specific subsets of TSH and FT4 instruments

No significant associations were found when the above analyses were stratified for specific subsets of TSH instruments (i.e. variants associated with AITD and variants not associated with AITD) and FT4 instruments (i.e. variants within the deiodinase loci and other (non-deiodinase) loci), as shown in Supplementary Tables 9 and 10. Neither did we find any associations when *DIO2* variants were analyzed separately (data not shown).

#### Causal effects of mood disorders on TSH and FT4 levels

To further investigate the relationship between thyroid function and mood disorders, we performed bidirectional MR analyses assessing the effects of mood disorders on TSH and FT4 levels. As shown in Figure 3, we observed no causal effects of genetic predisposition to MDD and BD on TSH and FT4 levels after conducting the reverse MR. Sensitivity analyses using the MR Egger and WM methods provided similar results. No evidence of directional pleiotropy was found based on the Egger intercept or using the MR-PRESSO method (Supplementary Table 11).

## Discussion

This is the first study using the MR approach to investigate the causal relationship between thyroid function and mood disorders. Given the availability of large GWAS datasets on thyroid function, MDD and BD, it was now the optimal moment to perform well-powered MR analyses on these endpoints (51). We found no evidence of causal effects of variation in normal-range thyroid function on MDD risk. Conversely, we observed a nominally significant inverse association between normal-range FT4 levels and BD risk.

Although the epidemiological association between variation in normal-range thyroid function and MDD risk has been reported in several population-based studies (12–14), we found no evidence of causal effects of variation in normal-range TSH or FT4 levels on MDD risk in our study. Due to availability of large datasets, we had sufficient power to even detect a 2.7% and 3.8% change in MDD risk per one SD change in TSH and FT4 levels, respectively. Therefore, the negative results of our study show that variation in normal-range thyroid function either does not affect MDD risk at all or, if present, the causal effects are much smaller than those reported in observational studies (12–14). As thyroid function might also affect the risk of minor forms of depression, we also tested the risk of depressive symptoms in subjects not meeting formal MDD criteria in order to cover the entire spectrum of depressive disorders, but neither found any associations. Importantly, there is strong evidence for associations between various autoimmune disorders and MDD (46–49). As AITD is the most common cause of thyroid dysfunction in the general population, the association between thyroid function tests and MDD reported in observational studies could be driven by the underlying autoimmunity or association with other autoimmune diseases instead of by thyroid function itself. Nonetheless, analyzing the two subsets of TSH associated variants (*i.e.* AITD and non-AITD associated variants) to separate thyroid from potential autoimmunity-mediated effects, resulted in no associations with MDD for any of the two subsets of instruments. We neither found any evidence of causal effects of MDD on TSH and FT4 levels to support reverse causality. Finally, potential non-linear associations between normal-range thyroid function and MDD risk could explain the absence of associations in our MR analyses. However, this is also unlikely since two large population-based studies investigated the association between normal-range TSH levels and MDD and did not find evidence for a U-shaped association (13, 14). Taken together, our results strongly indicate that variation in normal-range thyroid function does not have any noteworthy causal effect on the risk of MDD.

In our study, we observed a nominally significant inverse association between FT4 levels and BD risk, as well as BD1 risk. Although these findings were not statistically significant after multiple testing correction, they are in line with the results of observational and experimental studies suggesting beneficial effects of adjunct treatment with thyroid hormones in euthyroid patients with refractory BD (25, 26, 52, 53). Several underlying mechanisms may explain these findings, including increased serotonin neurotransmission (54), and modulation of the β-adrenergic receptor response to catecholamines in the brain (55). Interestingly, it has also been hypothesized that high dose levothyroxine (LT4) therapy may correct for cellular hypothyroidism in brain tissue in patients with BD (56). This hypothesis is based on an observation that BD is associated with mitochondrial dysfunction which results in low cellular adenosine triphosphate (ATP) levels (57, 58). As the cellular uptake of thyroid hormones is a transporter-mediated and energy-dependent process, inadequate ATP levels may disturb the intracellular transport of thyroid hormones, leading to cellular hypothyroidism (59). However, it has been shown that chronic energy depravation does not decrease the uptake of thyroid hormones by the anterior pituitary, as it does in the liver and other tissues (60, 61). This may result in a condition where the blood and pituitary levels of thyroid hormones are normal but they are low in other tissues, including neuron cells. In such a situation, high dose thyroid hormone therapy may facilitate the intracellular transport of thyroid hormones and correct cellular hypothyroidism (56). Indeed, next to the clinical studies showing beneficial effects of LT4 supplementation on depressive symptoms in BD, also functional imaging studies show that LT4 therapy corrects abnormal brain physiology in BD (26, 52). Thus, genetically determined higher FT4 levels could act in a similar way as LT4 supplementation, resulting in decreased BD risk. In line with this hypothesis, we found no evidence of association between genetically predicted TSH levels and BD risk, which suggests that the observed effects of genetically determined higher FT4 levels within the normal range on BD risk result from altered local FT4 bioavailability rather than a classical hyperthyroid-like state, which would coincide with decreased TSH levels. Although the described underlying mechanism for the observed associations seems plausible, further research is needed to confirm this hypothesis. Finally, BD patients are often treated with lithium, which can affect thyroid function (62). However, as the prevalence of lithium treatment in the general population is low, its effects on the results of GWAS on TSH and FT4 levels used in our MR study are negligible and therefore cannot explain the observed association between genetically predicted FT4 levels and BD.

Thyroid function tests are the first-line laboratory examinations in patients diagnosed with MDD, since symptoms of overt thyroid disease can mimic depression (63). However, it is still currently unknown if subclinical thyroid dysfunction in MDD patients should be treated or not, which is a common clinical problem in daily psychiatric practice, as subclinical hypo- and hyperthyroidism are prevalent, affecting 5-15% of the general population (64). As our genetic variants do not only associate with normal-range thyroid function, but a genetic risk score based on our TSH-associated variants also strongly associates with both subclinical hypo- and hyperthyroidism (35), the results of the current study suggest that there is neither any noteworthy causal effect of subclinical thyroid dysfunction on MDD risk. While in contrast to some previous observational studies (8), this is in line with the results of a recent large-scale (N=23,038) analysis of individual-level data from several prospective cohorts (65).

Up to a third of BD patients do not respond adequately to standard treatments (66). While LT4 therapy in BD patients remains controversial (24), our study provides novel evidence that supports the rationale for such a treatment. This is important, since LT4 treatment could be a valuable, low-cost and well-titratable therapeutic option for patients with treatment resistant BD. Therefore, our results should encourage further clinical studies on thyroid hormone therapy in BD.

This is a well-powered study that could detect even smaller effects of variation in normal-range thyroid function on the tested outcomes than those reported in current literature (12–14). As we used the MR approach and tested the robustness of our findings with several complementary statistical methods aimed to account for any pleiotropic instruments, the associations observed in our study are not affected by any residual confounders (29). We used genetic instruments which have in previous MR studies proven to be able to successfully identify causal associations between thyroid function and various classical thyroid hormone dependent endpoints including cholesterol levels, blood pressure, atrial fibrillation and stroke (30–32). In our secondary analyses, we assessed the associations between thyroid function and specific MDD and BD subtypes as well as minor depressive symptoms, thereby testing the entire spectrum of depressive disorders. We also performed a comprehensive assessment of complex relations between thyroid function and mood disorders, as we investigated the reverse causation in bidirectional MR analyses, and we took the potential autoimmunity-mediated effects of the used instruments in our sensitivity analyses into account.

A potential limitation of our study is the restriction to individuals of European ancestry, which means that our findings should not be directly extrapolated to other populations. Finally, since a well-powered GWAS on triiodothyronine (T3) levels is still lacking, we did not perform MR analyses with T3 as the exposure, which could have provided additional insight into the observed causal association between FT4 levels and BD.

In conclusion, we show that minor variations in TSH and FT4 levels have no effects on the risk of MDD and its subtypes, and neither on minor depressive symptoms. This is clinically relevant as it also suggests that depressive symptoms in patients with MDD should not be attributed to minor alterations in their thyroid function. In contrast, we show that higher FT4 levels within the normal range might be associated with lower BD risk, which is in line with the results of observational and experimental studies suggesting beneficial effects of FT4 treatment in patients with BD. These results should encourage further studies on thyroid hormone therapy in BD, including a large and well-powered RCT.

## Supplementary Material

Supplementary materials and methods

Supplementary tables
