# Supplementary material for: Thyroid function and mood disorders: a Mendelian Randomization study
Source: Thyroid. Author manuscript; Available in PMC 2022 Jul 6. (PMC7612998; doi:10.1089/thy.2020.0884)
Supplement: Supplementary materials and methods [file EMS146246-supplement-Supplementary_materials_and_methods.doc]

**Thyroid function and mood disorders: a Mendelian Randomization study**

Aleksander Kuś, Alisa D. Kjaergaard, Eirini Marouli, Fabiola Del Greco M., Rosalie B.T.M. Sterenborg, Layal Chaker, Robin P. Peeters, Tomasz Bednarczuk, Bjørn O. Åsvold, Stephen Burgess, Panos Deloukas, Alexander Teumer, Christina Ellervik, Marco Medici

**Supplementary Materials and Methods**

**Main analyses**

The main analyses included two-sample MR analyses performed using the inverse-variance weighted (IVW) method . This approach requires several assumptions of which the most important are that: (i) the genetic variants used as instruments have to be truly associated with the exposure (*i.e.* TSH or FT4 levels), and (ii) the effect of the instruments on the outcome of interest (*i.e.* MDD or BD) has to be mediated solely by the exposure under study . This means that weak and pleiotropic instruments should be avoided as they can strongly bias the causal estimates . To this end, we assessed the strength of all instruments based on the F statistics estimated as F=β^2^_exposure_/SE^2^_exposure_, which indicated no weak instruments (F statistics ranged 29.81-535.70 and 30.25-455.33 for the TSH and FT4 instruments, respectively), and we addressed the problem of potential pleiotropy in the sensitivity analyses.

**Sensitivity analyses**

Sensitivity analyses were performed in order to account for potential pleiotropy in the associations between thyroid function and the outcomes of interest. First, we compared the results obtained using the IVW method with the results from the MR Egger and weighted median (WM) methods, as the slope of the MR Egger regression may provide valid MR estimates in the presence of horizontal pleiotropy when the pleiotropic effects of the genetic variants are independent from the genetic associations with the exposure , while WM can provide valid MR estimates under the presence of horizontal pleiotropy when up to half of the included instruments are invalid . Egger intercept was also used as one of the indicators of directional pleiotropy . Furthermore, we also used the Mendelian Randomization Pleiotropy RESidual Sum and Outlier (MR-PRESSO) method to identify potentially pleiotropic variants and to correct for horizontal pleiotropy via outlier removal . Finally, several variants associated with TSH levels in the GWAS by Teumer *et al.* have also been associated with autoimmune thyroid disease (AITD), including Hashimoto's thyroiditis, Graves' disease, and thyroid peroxidase antibody positivity . As autoimmunity in general has been associated with mood disorders , we identified two separate subsets of TSH associated variants (*i.e.* variants associated with AITD and variants not associated with AITD analyzed separately) in order to separate potential thyroid from autoimmunity mediated effects. Moreover, as the genetic variants associated with FT4 levels form a highly heterogeneous group with potentially diverse effects on T4 and T3 bioavailability , we also identified two separate subsets of FT4 associated variants, specifically including: (i) variants within the deiodinases loci (*i.e.* *DIO1* and *DIO2*), and (ii) other (non-deiodinase) genetic variants associated with FT4 levels in the GWAS by Teumer *et al*. .

**Reverse MR analyses**

Reverse MR analyses on TSH and FT4 levels and MDD and BD were performed to gain insight into the complex and potentially bidirectional associations between thyroid function and mood disorders. A list of genetic variants associated with MDD and BD at a genome-wide significant level (P<5.0x10^-8^) and corresponding summary statistics were derived from the study by Howard *et al*. and Stahl *et al*. , respectively. Effect estimates on TSH and FT4 levels for these variants were derived from the GWAS on thyroid function performed by Teumer *et al.* , available at the ThyroidOmics Consortium website (www.thyroidomics.com).

**Power calculations**

To estimate the power of our study, we used a non-centrality parameter-based approach , implemented in a publicly available mRnd web tool (http://cnsgenomics.com/shiny/mRnd/). We calculated minimal odds ratio (OR) of the outcome variable per standard deviation (SD) of the exposure variable (TSH and FT4 levels) that was detectable (power=0.8, α=0.05) in our study. Proportions of total variance in TSH and FT4 levels explained by the genetic variants used as instruments (9.4% and 4.8%, respectively) were established based on the data from Teumer *et al.* . The results of power calculations are provided in **Supplementary Table 5**.

**References**

**1.** Burgess S, Butterworth A, Thompson SG 2013 Mendelian randomization analysis with multiple genetic variants using summarized data. Genet Epidemiol **37**:658-665.

**2.** Burgess S, Bowden J, Fall T, Ingelsson E, Thompson SG 2017 Sensitivity Analyses for Robust Causal Inference from Mendelian Randomization Analyses with Multiple Genetic Variants. Epidemiology **28**:30-42.

**3.** Burgess S, Thompson SG, Collaboration CCG 2011 Avoiding bias from weak instruments in Mendelian randomization studies. Int J Epidemiol **40**:755-764.

**4.** Glymour MM, Tchetgen Tchetgen EJ, Robins JM 2012 Credible Mendelian randomization studies: approaches for evaluating the instrumental variable assumptions. Am J Epidemiol **175**:332-339.

**5.** Bowden J, Davey Smith G, Burgess S 2015 Mendelian randomization with invalid instruments: effect estimation and bias detection through Egger regression. Int J Epidemiol **44**:512-525.

**6.** Bowden J, Davey Smith G, Haycock PC, Burgess S 2016 Consistent Estimation in Mendelian Randomization with Some Invalid Instruments Using a Weighted Median Estimator. Genet Epidemiol **40**:304-314.

**7.** Verbanck M, Chen CY, Neale B, Do R 2018 Detection of widespread horizontal pleiotropy in causal relationships inferred from Mendelian randomization between complex traits and diseases. Nat Genet **50**:693-698.

**8.** Teumer A, Chaker L, Groeneweg S, Li Y, Di Munno C, Barbieri C, Schultheiss UT, Traglia M, Ahluwalia TS, Akiyama M, Appel EVR, Arking DE, Arnold A, Astrup A, Beekman M, Beilby JP, Bekaert S, Boerwinkle E, Brown SJ, De Buyzere M, Campbell PJ, Ceresini G, Cerqueira C, Cucca F, Deary IJ, Deelen J, Eckardt KU, Ekici AB, Eriksson JG, Ferrrucci L, Fiers T, Fiorillo E, Ford I, Fox CS, Fuchsberger C, Galesloot TE, Gieger C, Gogele M, De Grandi A, Grarup N, Greiser KH, Haljas K, Hansen T, Harris SE, van Heemst D, den Heijer M, Hicks AA, den Hollander W, Homuth G, Hui J, Ikram MA, Ittermann T, Jensen RA, Jing J, Jukema JW, Kajantie E, Kamatani Y, Kasbohm E, Kaufman JM, Kiemeney LA, Kloppenburg M, Kronenberg F, Kubo M, Lahti J, Lapauw B, Li S, Liewald DCM, Lifelines Cohort S, Lim EM, Linneberg A, Marina M, Mascalzoni D, Matsuda K, Medenwald D, Meisinger C, Meulenbelt I, De Meyer T, Meyer Zu Schwabedissen HE, Mikolajczyk R, Moed M, Netea-Maier RT, Nolte IM, Okada Y, Pala M, Pattaro C, Pedersen O, Petersmann A, Porcu E, Postmus I, Pramstaller PP, Psaty BM, Ramos YFM, Rawal R, Redmond P, Richards JB, Rietzschel ER, Rivadeneira F, Roef G, Rotter JI, Sala CF, Schlessinger D, Selvin E, Slagboom PE, Soranzo N, Sorensen TIA, Spector TD, Starr JM, Stott DJ, Taes Y, Taliun D, Tanaka T, Thuesen B, Tiller D, Toniolo D, Uitterlinden AG, Visser WE, Walsh JP, Wilson SG, Wolffenbuttel BHR, Yang Q, Zheng HF, Cappola A, Peeters RP, Naitza S, Volzke H, Sanna S, Kottgen A, Visser TJ, Medici M 2018 Genome-wide analyses identify a role for SLC17A4 and AADAT in thyroid hormone regulation. Nat Commun **9**:4455.

**9.** Brcic L, Baric A, Gracan S, Brekalo M, Kalicanin D, Gunjaca I, Torlak Lovric V, Tokic S, Radman M, Skrabic V, Miljkovic A, Kolcic I, Stefanic M, Glavas-Obrovac L, Lessel D, Polasek O, Zemunik T, Barbalic M, Punda A, Boraska Perica V 2019 Genome-wide association analysis suggests novel loci for Hashimoto's thyroiditis. J Endocrinol Invest **42**:567-576.

**10.** Medici M, Porcu E, Pistis G, Teumer A, Brown SJ, Jensen RA, Rawal R, Roef GL, Plantinga TS, Vermeulen SH, Lahti J, Simmonds MJ, Husemoen LL, Freathy RM, Shields BM, Pietzner D, Nagy R, Broer L, Chaker L, Korevaar TI, Plia MG, Sala C, Volker U, Richards JB, Sweep FC, Gieger C, Corre T, Kajantie E, Thuesen B, Taes YE, Visser WE, Hattersley AT, Kratzsch J, Hamilton A, Li W, Homuth G, Lobina M, Mariotti S, Soranzo N, Cocca M, Nauck M, Spielhagen C, Ross A, Arnold A, van de Bunt M, Liyanarachchi S, Heier M, Grabe HJ, Masciullo C, Galesloot TE, Lim EM, Reischl E, Leedman PJ, Lai S, Delitala A, Bremner AP, Philips DI, Beilby JP, Mulas A, Vocale M, Abecasis G, Forsen T, James A, Widen E, Hui J, Prokisch H, Rietzschel EE, Palotie A, Feddema P, Fletcher SJ, Schramm K, Rotter JI, Kluttig A, Radke D, Traglia M, Surdulescu GL, He H, Franklyn JA, Tiller D, Vaidya B, de Meyer T, Jorgensen T, Eriksson JG, O'Leary PC, Wichmann E, Hermus AR, Psaty BM, Ittermann T, Hofman A, Bosi E, Schlessinger D, Wallaschofski H, Pirastu N, Aulchenko YS, de la Chapelle A, Netea-Maier RT, Gough SC, Meyer Zu Schwabedissen H, Frayling TM, Kaufman JM, Linneberg A, Raikkonen K, Smit JW, Kiemeney LA, Rivadeneira F, Uitterlinden AG, Walsh JP, Meisinger C, den Heijer M, Visser TJ, Spector TD, Wilson SG, Volzke H, Cappola A, Toniolo D, Sanna S, Naitza S, Peeters RP 2014 Identification of novel genetic Loci associated with thyroid peroxidase antibodies and clinical thyroid disease. PLoS Genet **10**:e1004123.

**11.** Euesden J, Danese A, Lewis CM, Maughan B 2017 A bidirectional relationship between depression and the autoimmune disorders - New perspectives from the National Child Development Study. PLoS One **12**:e0173015.

**12.** Pryce CR, Fontana A 2017 Depression in Autoimmune Diseases. Curr Top Behav Neurosci **31**:139-154.

**13.** Siegmann EM, Muller HHO, Luecke C, Philipsen A, Kornhuber J, Gromer TW 2018 Association of Depression and Anxiety Disorders With Autoimmune Thyroiditis: A Systematic Review and Meta-analysis. JAMA Psychiatry **75**:577-584.

**14.** Benros ME, Waltoft BL, Nordentoft M, Ostergaard SD, Eaton WW, Krogh J, Mortensen PB 2013 Autoimmune diseases and severe infections as risk factors for mood disorders: a nationwide study. JAMA Psychiatry **70**:812-820.

**15.** Kus A, Chaker L, Teumer A, Peeters RP, Medici M 2020 The Genetic Basis of Thyroid Function: Novel Findings and New Approaches. J Clin Endocrinol Metab. doi: 10.1210/clinem/dgz225.

**16.** Medici M, Peeters RP, Teumer A, Taylor P 2019 The importance of high-quality mendelian randomisation studies for clinical thyroidology. Lancet Diabetes Endocrinol **7**:665-667.

**17.** Howard DM, Adams MJ, Clarke TK, Hafferty JD, Gibson J, Shirali M, Coleman JRI, Hagenaars SP, Ward J, Wigmore EM, Alloza C, Shen X, Barbu MC, Xu EY, Whalley HC, Marioni RE, Porteous DJ, Davies G, Deary IJ, Hemani G, Berger K, Teismann H, Rawal R, Arolt V, Baune BT, Dannlowski U, Domschke K, Tian C, Hinds DA, andMe Research T, Major Depressive Disorder Working Group of the Psychiatric Genomics C, Trzaskowski M, Byrne EM, Ripke S, Smith DJ, Sullivan PF, Wray NR, Breen G, Lewis CM, McIntosh AM 2019 Genome-wide meta-analysis of depression identifies 102 independent variants and highlights the importance of the prefrontal brain regions. Nat Neurosci **22**:343-352.

**18.** Stahl EA, Breen G, Forstner AJ, McQuillin A, Ripke S, Trubetskoy V, Mattheisen M, Wang Y, Coleman JRI, Gaspar HA, de Leeuw CA, Steinberg S, Pavlides JMW, Trzaskowski M, Byrne EM, Pers TH, Holmans PA, Richards AL, Abbott L, Agerbo E, Akil H, Albani D, Alliey-Rodriguez N, Als TD, Anjorin A, Antilla V, Awasthi S, Badner JA, Baekvad-Hansen M, Barchas JD, Bass N, Bauer M, Belliveau R, Bergen SE, Pedersen CB, Boen E, Boks MP, Boocock J, Budde M, Bunney W, Burmeister M, Bybjerg-Grauholm J, Byerley W, Casas M, Cerrato F, Cervantes P, Chambert K, Charney AW, Chen D, Churchhouse C, Clarke TK, Coryell W, Craig DW, Cruceanu C, Curtis D, Czerski PM, Dale AM, de Jong S, Degenhardt F, Del-Favero J, DePaulo JR, Djurovic S, Dobbyn AL, Dumont A, Elvsashagen T, Escott-Price V, Fan CC, Fischer SB, Flickinger M, Foroud TM, Forty L, Frank J, Fraser C, Freimer NB, Frisen L, Gade K, Gage D, Garnham J, Giambartolomei C, Pedersen MG, Goldstein J, Gordon SD, Gordon-Smith K, Green EK, Green MJ, Greenwood TA, Grove J, Guan W, Guzman-Parra J, Hamshere ML, Hautzinger M, Heilbronner U, Herms S, Hipolito M, Hoffmann P, Holland D, Huckins L, Jamain S, Johnson JS, Jureus A, Kandaswamy R, Karlsson R, Kennedy JL, Kittel-Schneider S, Knowles JA, Kogevinas M, Koller AC, Kupka R, Lavebratt C, Lawrence J, Lawson WB, Leber M, Lee PH, Levy SE, Li JZ, Liu C, Lucae S, Maaser A, MacIntyre DJ, Mahon PB, Maier W, Martinsson L, McCarroll S, McGuffin P, McInnis MG, McKay JD, Medeiros H, Medland SE, Meng F, Milani L, Montgomery GW, Morris DW, Muhleisen TW, Mullins N, Nguyen H, Nievergelt CM, Adolfsson AN, Nwulia EA, O'Donovan C, Loohuis LMO, Ori APS, Oruc L, Osby U, Perlis RH, Perry A, Pfennig A, Potash JB, Purcell SM, Regeer EJ, Reif A, Reinbold CS, Rice JP, Rivas F, Rivera M, Roussos P, Ruderfer DM, Ryu E, Sanchez-Mora C, Schatzberg AF, Scheftner WA, Schork NJ, Shannon Weickert C, Shehktman T, Shilling PD, Sigurdsson E, Slaney C, Smeland OB, Sobell JL, Soholm Hansen C, Spijker AT, St Clair D, Steffens M, Strauss JS, Streit F, Strohmaier J, Szelinger S, Thompson RC, Thorgeirsson TE, Treutlein J, Vedder H, Wang W, Watson SJ, Weickert TW, Witt SH, Xi S, Xu W, Young AH, Zandi P, Zhang P, Zollner S, e QC, Consortium B, Adolfsson R, Agartz I, Alda M, Backlund L, Baune BT, Bellivier F, Berrettini WH, Biernacka JM, Blackwood DHR, Boehnke M, Borglum AD, Corvin A, Craddock N, Daly MJ, Dannlowski U, Esko T, Etain B, Frye M, Fullerton JM, Gershon ES, Gill M, Goes F, Grigoroiu-Serbanescu M, Hauser J, Hougaard DM, Hultman CM, Jones I, Jones LA, Kahn RS, Kirov G, Landen M, Leboyer M, Lewis CM, Li QS, Lissowska J, Martin NG, Mayoral F, McElroy SL, McIntosh AM, McMahon FJ, Melle I, Metspalu A, Mitchell PB, Morken G, Mors O, Mortensen PB, Muller-Myhsok B, Myers RM, Neale BM, Nimgaonkar V, Nordentoft M, Nothen MM, O'Donovan MC, Oedegaard KJ, Owen MJ, Paciga SA, Pato C, Pato MT, Posthuma D, Ramos-Quiroga JA, Ribases M, Rietschel M, Rouleau GA, Schalling M, Schofield PR, Schulze TG, Serretti A, Smoller JW, Stefansson H, Stefansson K, Stordal E, Sullivan PF, Turecki G, Vaaler AE, Vieta E, Vincent JB, Werge T, Nurnberger JI, Wray NR, Di Florio A, Edenberg HJ, Cichon S, Ophoff RA, Scott LJ, Andreassen OA, Kelsoe J, Sklar P, Bipolar Disorder Working Group of the Psychiatric Genomics C 2019 Genome-wide association study identifies 30 loci associated with bipolar disorder. Nat Genet **51**:793-803.

**19.** Brion MJ, Shakhbazov K, Visscher PM 2013 Calculating statistical power in Mendelian randomization studies. Int J Epidemiol **42**:1497-1501.
