# Supplementary material for: Thyroid function and mood disorders: a Mendelian Randomization study
Source: Thyroid. Author manuscript; Available in PMC 2022 Jul 6. (PMC7612998; doi:10.1089/thy.2020.0884)
Supplement: Supplementary tables [file EMS146246-supplement-Supplementary_tables.pdf]

Supplementary Tables (STables) Index

**STable 1**	Datasets for the tested outcomes used in the Mendelian Randomization (MR) analyses.
**STable 2**	Phenotype definitions for the tested outcomes.
**STable 3**	Variants used as instruments for Mendelian Randomization analyses with normal range TSH levels as the exposure.
**STable 4**	Variants used as instruments for Mendelian Randomization analyses with normal range FT4 levels as the exposure.
**STable 5**	Power calculations.
**STable 6**	Results of the Mendelian Randomization (MR) analyses investigating the association between genetically predicted TSH and FT4 levels and the tested outcomes using various MR methods.
**STable 7**	Intercept values from the Mendelian Randomization analyses investigating the association between genetically predicted TSH and FT4 levels and the tested outcomes using the MR Egger method.
**STable 8**	Detailed results of the Mendelian Randomization (MR) analyses investigating the association between genetically predicted TSH and FT4 levels and the tested outcomes using the MR PRESSO method.
**STable 9**	Results of the Mendelian Randomization (MR) analyses using as instruments two specific subsets of TSH associated variants.
**STable 10**	Results of the Mendelian Randomization (MR) analyses using as instruments two specific subsets of FT4 associated variants.
**STable 11**	Results of the Mendelian Randomization analyses investigating the effects of MDD and BD on TSH and FT4 levels.

Supplementary Table 1. Datasets for the tested outcomes used in the Mendelian randomization (MR) analyses.

Outcome	Abbreviation	Ncases	Ncontrols	Consortium	Reference PMID
**Major Depressive Disorder**	**MDD**	170.756	329.443	PGC+UKBB	30718901
recurent MDD	rMDD	17.451	63.482	UKBB	31926635
single-episode MDD	sMDD	12.024	63.482	UKBB	31926635
sub-threshold MDD	subMDD	21.596	63.482	UKBB	31926635
**Bipolar Disorder**	**BD**	20.352	31.358	PGC	31043756
Bipolar Disorder type 1	BD1	14.879	30.992	PGC	31043756
Bipolar Disorder type 2	BD2	3.421	22.155	PGC	31043756
Schizoaffective Bipolar Disorder	SABD	977	8,690	PGC	31043756

Abbreviations: PGC, Psychiatric Genomics Consortium; UKBB, UK Biobank; PMID, PubMed Identifier.

Supplementary Table 2. Phenotype definitions for the tested outcomes.

A. Major depressive disorder (MDD)

Summary statistics for MDD were derived from the GWAS meta-analysis by Howard et al. (PMID: 30718901). This study meta-analyzed the results of previous GWAS on MDD in PGC, UKBB and 23andMe cohorts. As summary statistics including the 23andMe data are not publically available, we used the summary statistics from a meta-analysis of the 33 cohorts of the PGC and the broad depression phenotype in the UKBB. The total number of individuals in this data is 500,199 (170,756 cases and 329,443 controls). The detailed inclusion/exclusion criteria for cases and controls for each of the PGC cohorts are available in the original manuscript by Wray et al. (PMID: 29700475). Criteria for defining case and control status for the broad depression phenotype in the UKBB cohort are presented below. Square brackets are UK Biobank field identifiers.

Phenotype	Criteria for case	Criteria for control	Exclusions
MDD	From touchscreen questionnaire - answered YES to at least one of the questions:<br>Have you ever seen a general practitioner for nerves, anxiety, tension or depression? [f2090]<br>Have you ever seen a psychiatrist for nerves, anxiety, tension or depression? [f2100]<br><br>OR<br>From Hospital Episodes Data from UK bodies (English HES Data, Scottish Morbidity Register, Patient Episode Data):<br>Any primary [f41202] or secondary [f41204] diagnosis of ICD-10 Codes for mood disorders:<br>F32 - Single Episode Depression<br>F33 - Recurrent Depression<br>F34 - Persistent mood disorders (Cyclothymia, Dysthymia)<br>F38 - Other mood disorders<br>F39 - Unspecified mood disorders	From touchscreen questionnaire - answered NO to both questions:<br>Have you ever seen a general practitioner for nerves, anxiety, tension or depression? [f2090]<br>Have you ever seen a psychiatrist for nerves, anxiety, tension or depression? [f2100]	participants identified with bipolar disorder, schizophrenia, or personality disorder using self-declared data following the approach of Smith et al. (PMID: 24282498)<br>participants with prescriptions for antipsychotic medications

B. MDD subtypes.

Criteria for defining case and control status for MDD subtypes in the UK Biobank Mental Health Questionnaire are presented below. Square brackets are UK Biobank field identifiers. Adapted from Coleman et al. (PMID: 31926635).
In brief, individuals meeting criteria for major depressive disorder were classified as "recurrent" if they reported multiple depressed periods across their lifetime (rMDD, N = 17,451), and "single-episode" otherwise (sMDD, N = 12,024).
Individuals reporting depressive symptoms but not meeting case criteria were excluded from MDD but used as a "sub-threshold depression" subtype to examine the continuity of genetic associations with major depressive disorder below clinical thresholds (subMDD, N = 21,596).

Phenotype	Criteria for case	Criteria for control	Exclusions
MDD	Report 5 of the below (at least one of the bold items):<br>**Ever had prolonged feelings of sadness or depression [f20446]**<br>**Ever had prolonged loss of interest in normal activities [f20441]**<br>Feelings of tiredness during worst episode of depression [f20449]<br>Weight change during worst episode of depression [f20536]<br>Sleep change during worst episode of depression [f20532]<br>Difficulty concentrating during worst episode of depression [f20435]<br>Feelings of worthlessness during worst episode of depression [f20450]<br>Thoughts of death during worst episode of depression [f20437]<br><br>AND report<br>Most of the day or more affected during worst episode of depression [f20436]<br>Depressed (almost) every day during worst episode of depression [f20439]<br>More than a little impact on normal roles during worst period of depression [f20440]	Do not:<br>Meet case status<br>Report any mental health problems diagnosed by a professional [f20544]<br>Report depression in previous interview with psychiatric nurse [f20002]<br>Meet previous criteria for depression or bipolar disorder [f20126]<br>Have a hospital inpatient ICD10 code for mood disorder (F30-F39) [f.41202 / f.41204]<br>Report use of anti-depressant medication* at baseline [f.20003]<br>Report extensive recent symptoms of depression: less than 14 on summed response (where "not at all" = 1 and "nearly every day" = 4) to recent<br>Lack of interest or pleasure in doing things [f20514]<br>Feelings of inadequacy [f20507]<br>Feelings of depression [f20510]<br>Trouble concentrating on things [f20508]<br>Changes in speed or amount of moving or speaking [f20518]<br>Feelings of tiredness or low energy [f20519]<br>Thoughts of suicide or self-harm [f20513]<br>Poor appetite or overeating [f20511]	Report any of the following mental health problems diagnosed by a professional [f20544]:<br>Mania, hypomania, bipolar or manic-depression<br>Schizophrenia<br>Any other type of psychosis or psychotic illness
rMDD	MDD and:<br>Lifetime number of depressed periods [f20442] > 1		
sMDD	MDD and:<br>Lifetime number of depressed periods [f20442] = 1		
subMDD	Individuals reporting depressive symptoms but not meeting MDD criteria		

C. Bipolar disorder (BD) and BD subtypes.

Summary statistics for BD and BD subtypes (BD1, BD2, SABD) were derived from the GWAS meta-analysis of 32 studies from 14 countries in Europe, North America and Australia, totaling 20,352 cases and 31,358 controls of European descent.
Cases were required to meet international consensus criteria (DSM-IV, ICD-9, or ICD-10) for a lifetime diagnosis of BD established using structured diagnostic instruments from assessments by trained interviewers, clinician-administered checklists, or medical record review.
Controls in most samples were screened for the absence of lifetime psychiatric disorders. The detailed inclusion/exclusion criteria for cases and controls for each study included in the meta-analysis are available in the original manuscript by Stahl et al. (PMID: 31043756).

Supplementary Table 3. Variants used as instruments for Mendelian Randomization analyses with normal range TSH levels as the exposure.

Chr	Pos	RSID	GeneName	Effect_allele	Other_allele	EAF_TSH	Effect_TSH	StdErr_TSH	Pvalue_TSH	Fstat	logOR_MDD	StdErr_MDD	Pvalue_MDD	logOR_rMDD	StdErr_rMDD	Pvalue_rMDD	logOR_sMDD	StdErr_sMDD	Pvalue_sMDD	logOR_subMDD	StdErr_subMDD	Pvalue_subMDD	logOR_BD	StdErr_BD	Pvalue_BD	logOR_BD1	StdErr_BD1	Pvalue_BD1	logOR_BD2	StdErr_BD2	Pvalue_BD2	logOR_SABD	StdErr_SABD	Pvalue_SABD
1	19771438	rs12089835	CAPZB	t	c	0.3479	0.0725	0.0065	1.27E-28	124.41	0.0063	0.0045	0.1582	-0.0080	0.0207	0.7008	-0.0417	0.0272	0.1253	0.0097	0.0181	0.5911	-0.0202	0.0140	0.1495	-0.0119	0.0157	0.4496	-0.0466	0.0286	0.1032	-0.0387	0.0533	0.4682
1	19843576	rs10917469	CAPZB	a	g	0.8439	0.1112	0.0085	3.95E-39	171.15	-0.0053	0.0059	0.3764	-0.0146	0.0208	0.4828	-0.0479	0.0270	0.0759	-0.0205	0.0182	0.2599	-0.0023	0.0185	0.9001	0.0054	0.0208	0.7964	-0.0546	0.0376	0.1462	-0.0509	0.0702	0.4682
1	19862320	rs74804879	CAPZB	t	c	0.6486	0.0501	0.0065	1.22E-14	59.41	-0.0019	0.0046	0.6823	NA	NA	NA	NA	NA	NA	NA	NA	NA	-0.0070	0.0142	0.6210	-0.0076	0.0159	0.6356	0.0009	0.0288	0.9757	-0.0223	0.0544	0.6823
1	61610049	rs334725	NFIA	a	g	0.9520	0.1737	0.0147	2.45E-32	139.63	-0.0100	0.0103	0.3355	NA	NA	NA	NA	NA	NA	NA	NA	NA	0.0054	0.0328	0.8705	NA	NA	NA	NA	NA	NA	NA	NA	NA
1	108357391	rs17020122	VAV3	t	c	0.0852	0.1044	0.0114	5.32E-20	83.87	0.0043	0.0075	0.5687	0.0311	0.0209	0.1359	0.0270	0.0273	0.3229	0.0189	0.0183	0.3019	-0.0495	0.0236	0.0358	-0.0537	0.0268	0.0449	-0.0383	0.0468	0.4132	-0.0835	0.0890	0.3484
2	217580413	rs16856540	IGFBP5	t	c	0.8381	-0.0549	0.0084	7.81E-11	42.72	-0.0067	0.0060	0.2671	-0.0065	0.0209	0.7556	-0.0234	0.0273	0.3905	-0.0069	0.0182	0.7041	-0.0186	0.0188	0.3222	-0.0291	0.0210	0.1669	0.0093	0.0382	0.8075	0.0171	0.0711	0.8096
2	217625523	rs13015993	IGFBP5	a	g	0.7333	0.0818	0.0069	4.52E-32	140.54	-0.0078	0.0048	0.1089	-0.0269	0.0209	0.1983	0.0293	0.0276	0.2876	0.0180	0.0183	0.3249	-0.0392	0.0151	0.0092	-0.0343	0.0170	0.0436	-0.0366	0.0303	0.2272	-0.0659	0.0574	0.2507
2	218236786	rs6724073	DIRC3	t	c	0.7406	0.0508	0.0079	1.35E-10	41.35	-0.0041	0.0051	0.4242	0.0023	0.0209	0.9141	-0.0134	0.0272	0.6239	0.0277	0.0183	0.1305	-0.0053	0.0157	0.7339	-0.0032	0.0176	0.8572	-0.0013	0.0315	0.9670	0.0294	0.0583	0.6137
3	12239852	rs1663070	SYN2	t	c	0.7417	-0.0463	0.0070	3.49E-11	43.75	-0.0120	0.0050	0.0158	-0.0349	0.0209	0.0947	-0.0630	0.0272	0.0206	-0.0217	0.0183	0.2349	-0.0378	0.0154	0.0142	-0.0443	0.0173	0.0105	-0.0168	0.0312	0.5902	-0.0211	0.0592	0.7221
3	149220109	rs28502438	TM4SF4	t	c	0.5668	0.0338	0.0061	3.70E-08	30.70	0.0026	0.0044	0.5443	-0.0008	0.0209	0.9690	-0.0082	0.0273	0.7634	0.0299	0.0182	0.1005	-0.0019	0.0138	0.8882	-0.0066	0.0155	0.6713	0.0073	0.0281	0.7938	0.0349	0.0525	0.5065
3	185514088	rs13100823	IGF2BP2	t	c	0.3061	-0.0406	0.0066	6.76E-10	37.84	0.0020	0.0047	0.6718	0.0366	0.0209	0.0803	0.0159	0.0275	0.5629	0.0107	0.0183	0.5601	0.0173	0.0145	0.2345	0.0112	0.0164	0.4948	0.0328	0.0294	0.2633	0.0588	0.0542	0.2777
3	193916181	rs59381142	HES1	a	g	0.2430	-0.0580	0.0076	1.70E-14	58.24	0.0028	0.0051	0.5854	0.0212	0.0208	0.3079	-0.0172	0.0273	0.5287	0.0156	0.0181	0.3884	-0.0195	0.0165	0.2385	-0.0132	0.0186	0.4770	-0.0457	0.0342	0.1807	-0.0193	0.0634	0.7603
4	149587905	rs6535624	NR3C2	a	g	0.4409	0.0419	0.0062	1.60E-11	45.67	-0.0067	0.0043	0.1224	-0.0446	0.0209	0.0330	-0.0060	0.0273	0.8256	-0.0201	0.0182	0.2681	0.0197	0.0135	0.1450	0.0346	0.0152	0.0230	-0.0276	0.0275	0.3158	-0.0349	0.0517	0.4994
4	149665602	rs11732089	NR3C2	t	c	0.7961	0.1150	0.0076	1.73E-51	228.96	0.0031	0.0053	0.5574	-0.0085	0.0209	0.6837	0.0028	0.0274	0.9181	0.0213	0.0183	0.2453	-0.0215	0.0170	0.2048	-0.0087	0.0191	0.6475	-0.0799	0.0342	0.0196	-0.0649	0.0639	0.3099
5	76439961	rs62362610	PDE8B	c	g	0.0830	0.0726	0.0118	7.73E-10	37.85	-0.0219	0.0076	0.0039	-0.0156	0.0210	0.4564	-0.0131	0.0274	0.6332	0.0127	0.0181	0.4818	-0.0342	0.0252	0.1741	-0.0360	0.0284	0.2055	-0.0302	0.0510	0.5535	-0.0021	0.0935	0.9820
5	76488613	rs1119208	PDE8B	t	c	0.3666	0.0457	0.0064	6.65E-13	50.99	-0.0012	0.0045	0.7842	0.0141	0.0208	0.4967	-0.0023	0.0272	0.9328	0.0034	0.0181	0.8527	-0.0189	0.0140	0.1750	-0.0157	0.0157	0.3176	0.0013	0.0283	0.9626	-0.0568	0.0534	0.2869
5	76495539	rs139424329	PDE8B	a	g	0.0140	-0.2000	0.0322	5.14E-10	38.58	0.0245	0.0220	0.2645	NA	NA	NA	NA	NA	NA	NA	NA	NA	-0.0616	0.0675	0.3619	NA	NA	NA	NA	NA	NA	NA	NA	NA
5	76532571	rs2127387	PDE8B	a	g	0.4087	0.1435	0.0062	1.10E-117	535.70	-0.0011	0.0044	0.7997	0.0233	0.0210	0.2685	0.0137	0.0275	0.6186	0.0157	0.0183	0.3919	0.0042	0.0137	0.7583	0.0153	0.0154	0.3197	0.0100	0.0278	0.7195	-0.0362	0.0516	0.4825
5	76554807	rs7702192	PDE8B	a	c	0.4723	0.0697	0.0061	2.61E-30	130.56	-0.0007	0.0043	0.8741	0.0048	0.0208	0.8160	0.0138	0.0272	0.6131	-0.0037	0.0181	0.8385	-0.0230	0.0134	0.0870	-0.0283	0.0151	0.0614	0.0207	0.0272	0.4481	-0.0290	0.0512	0.5714
5	76652403	rs113974964	PDE8B	t	c	0.0466	-0.1237	0.0146	2.06E-17	71.78	-0.0024	0.0104	0.8206	NA	NA	NA	NA	NA	NA	NA	NA	NA	-0.0020	0.0314	0.9480	NA	NA	NA	NA	NA	NA	NA	NA	NA
5	76660193	rs139149784	PDE8B	a	g	0.0271	0.1556	0.0285	4.97E-08	29.81	0.0269	0.0172	0.1181	NA	NA	NA	NA	NA	NA	NA	NA	NA	0.0401	0.0594	0.4994	NA	NA	NA	NA	NA	NA	NA	NA	NA
5	76773148	rs182873197	PDE8B	t	c	0.0511	-0.0799	0.0142	1.71E-08	31.66	0.0023	0.0102	0.8212	NA	NA	NA	NA	NA	NA	NA	NA	NA	0.0069	0.0307	0.8220	NA	NA	NA	NA	NA	NA	NA	NA	NA
6	31108129	rs1265091	PSORS1C1	t	c	0.2020	0.0571	0.0086	3.20E-11	44.08	NA	NA	NA	0.0081	0.0207	0.6959	-0.0130	0.0273	0.6342	0.0049	0.0181	0.7848	NA	NA	NA	NA	NA	NA	NA	NA	NA	NA	NA	NA
6	43805362	rs744103	VEGFA/LOC100132354	a	t	0.6909	0.0919	0.0069	6.73E-41	177.39	0.0013	0.0047	0.7832	0.0018	0.0211	0.9327	0.0181	0.0277	0.5143	-0.0089	0.0183	0.6292	-0.0121	0.0150	0.4184	-0.0020	0.0169	0.9042	-0.0520	0.0303	0.0864	0.1023	0.0579	0.0771
6	43905037	rs9381266	VEGFA/LOC100132354	t	c	0.7421	0.0726	0.0070	1.84E-25	107.57	-0.0006	0.0050	0.9086	-0.0423	0.0205	0.0395	-0.0265	0.0269	0.3248	0.0076	0.0180	0.6750	0.0052	0.0155	0.7382	0.0104	0.0174	0.5501	0.0028	0.0315	0.9299	-0.0705	0.0575	0.2207
6	148521292	rs9497965	SASH1	t	c	0.4007	0.0444	0.0062	9.81E-13	51.28	0.0074	0.0044	0.0933	-0.0261	0.0208	0.2099	-0.0248	0.0272	0.3615	0.0286	0.0181	0.1132	-0.0032	0.0138	0.8193	0.0091	0.0156	0.5601	-0.0374	0.0281	0.1821	0.0030	0.0524	0.9545
6	165973757	rs73022105	PDE10A	t	c	0.9548	0.1049	0.0155	1.20E-11	45.80	-0.0027	0.0109	0.8048	NA	NA	NA	NA	NA	NA	NA	NA	NA	0.0001	0.0332	0.9975	NA	NA	NA	NA	NA	NA	NA	NA	NA
6	166047034	rs1079418	PDE10A	a	g	0.6877	0.1009	0.0066	8.23E-53	233.72	0.0042	0.0047	0.3722	-0.0099	0.0208	0.6353	-0.0185	0.0272	0.4960	0.0129	0.0182	0.4759	-0.0066	0.0147	0.6542	0.0005	0.0165	0.9736	-0.0256	0.0298	0.3901	0.0265	0.0559	0.6358
8	23356964	rs56009477	SLC25A37	a	g	0.8383	0.0524	0.0084	3.72E-10	38.91	-0.0051	0.0059	0.3860	0.0218	0.0206	0.2909	-0.0285	0.0267	0.2857	-0.0183	0.0178	0.3036	0.0057	0.0187	0.7614	0.0187	0.0211	0.3750	-0.0678	0.0374	0.0702	0.0346	0.0711	0.6266
8	32433013	rs2439301	NRG1	a	g	0.2331	-0.0587	0.0076	8.15E-15	59.66	-0.0069	0.0052	0.1818	-0.0114	0.0209	0.5849	-0.0321	0.0275	0.2425	-0.0219	0.0183	0.2312	-0.0109	0.0165	0.5116	-0.0121	0.0186	0.5134	-0.0220	0.0337	0.5137	0.0520	0.0622	0.4029
8	70365025	rs10957494	SULF1	a	g	0.6915	-0.0402	0.0066	1.10E-09	37.10	0.0086	0.0047	0.0691	0.0103	0.0210	0.6242	-0.0084	0.0274	0.7598	0.0052	0.0183	0.7770	-0.0098	0.0146	0.5020	-0.0094	0.0164	0.5683	-0.0313	0.0298	0.2927	-0.0853	0.0554	0.1238
8	133771635	rs118039499	TG	a	c	0.9765	0.1837	0.0240	1.99E-14	58.59	-0.0108	0.0140	0.4392	NA	NA	NA	NA	NA	NA	NA	NA	NA	-0.0392	0.0477	0.4114	NA	NA	NA	NA	NA	NA	NA	NA	NA
8	133951991	rs2739067	TG	a	g	0.5980	-0.0415	0.0062	2.43E-11	44.80	0.0055	0.0044	0.2174	0.0223	0.0210	0.2881	0.0062	0.0274	0.8205	-0.0121	0.0182	0.5054	-0.0110	0.0138	0.4261	-0.0060	0.0155	0.6975	-0.0049	0.0280	0.8604	-0.0477	0.0521	0.3599
9	4290544	rs10814915	GLIS3	t	c	0.4438	0.0421	0.0061	5.06E-12	47.63	0.0009	0.0043	0.8284	-0.0079	0.0210	0.7071	-0.0408	0.0275	0.1378	0.0007	0.0183	0.9682	0.0017	0.0136	0.9013	0.0007	0.0153	0.9635	0.0080	0.0274	0.7706	-0.0421	0.0517	0.4156
9	16214340	rs9298749	C9orf92	a	c	0.5878	-0.0393	0.0064	8.80E-10	37.71	-0.0075	0.0045	0.0954	-0.0451	0.0208	0.0302	-0.0099	0.0273	0.7167	-0.0105	0.0182	0.5628	-0.0001	0.0141	0.9949	0.0122	0.0159	0.4428	-0.0498	0.0284	0.0790	-0.0474	0.0534	0.3750
10	8682180	rs11255790	GATA3	t	c	0.3020	-0.0410	0.0066	6.83E-10	38.59	-0.0045	0.0047	0.3403	0.0080	0.0207	0.6983	0.0237	0.0270	0.3801	0.0006	0.0180	0.9740	0.0197	0.0147	0.1809	0.0160	0.0166	0.3329	0.0534	0.0296	0.0711	-0.0104	0.0562	0.8536
10	89849519	rs4933466	PTEN	a	g	0.6045	0.0395	0.0063	5.13E-10	39.31	-0.0015	0.0046	0.7532	-0.0080	0.0208	0.7014	0.0192	0.0272	0.4802	-0.0096	0.0181	0.5954	-0.0005	0.0139	0.9693	0.0032	0.0157	0.8401	0.0085	0.0284	0.7654	-0.0303	0.0530	0.5673
10	101283330	rs200574439	NKX2-3	a	c	0.5760	-0.0467	0.0064	3.69E-13	53.24	NA	NA	NA	NA	NA	NA	NA	NA	NA	NA	NA	NA	NA	NA	NA	NA	NA	NA	NA	NA	NA	NA	NA	NA
11	45228686	rs12284404	PRDM11	a	g	0.2733	-0.0667	0.0069	2.48E-22	93.44	-0.0106	0.0047	0.0251	-0.0317	0.0207	0.1262	-0.0235	0.0271	0.3868	0.0004	0.0180	0.9833	0.0134	0.0148	0.3648	0.0132	0.0167	0.4286	0.0219	0.0297	0.4616	0.0544	0.0559	0.3301
11	115045237	rs4445669	CADM1	t	c	0.4591	-0.0397	0.0061	5.76E-11	42.36	0.0030	0.0043	0.4913	0.0040	0.0209	0.8500	0.0617	0.0273	0.0236	-0.0030	0.0182	0.8700	0.0023	0.0133	0.8639	-0.0011	0.0150	0.9407	0.0087	0.0271	0.7470	-0.0248	0.0509	0.6253
13	24782080	rs7329958	SPATA13	t	c	0.3482	-0.0439	0.0065	1.13E-11	45.61	-0.0042	0.0045	0.3527	-0.0111	0.0207	0.5918	0.0356	0.0270	0.1873	-0.0210	0.0181	0.2439	-0.0144	0.0142	0.3122	-0.0084	0.0160	0.6008	-0.0391	0.0290	0.1778	-0.0035	0.0541	0.9488
14	36536181	rs398745	MBIP	a	c	0.5943	-0.0520	0.0062	3.97E-17	70.34	-0.0009	0.0044	0.8304	0.0394	0.0209	0.0591	-0.0239	0.0272	0.3789	0.0075	0.0182	0.6809	-0.0266	0.0136	0.0496	-0.0295	0.0153	0.0537	-0.0218	0.0276	0.4303	0.0066	0.0517	0.8992
14	36713154	rs2254613	MBIP	t	g	0.5505	-0.0346	0.0063	3.44E-08	30.16	0.0008	0.0044	0.8629	0.0136	0.0209	0.5149	0.0178	0.0273	0.5126	0.0160	0.0181	0.3783	-0.0068	0.0136	0.6177	-0.0215	0.0153	0.1609	0.0070	0.0278	0.8017	0.0713	0.0512	0.1642
14	81490842	rs11159482	TSHR	t	c	0.0877	0.0846	0.0129	6.30E-11	43.01	0.0046	0.0091	0.6127	0.0275	0.0209	0.1885	-0.0293	0.0279	0.2946	-0.0083	0.0184	0.6529	0.0022	0.0267	0.9339	0.0240	0.0299	0.4213	-0.0517	0.0552	0.3493	-0.0831	0.1028	0.4188
14	81594143	rs59334515	TSHR	t	c	0.2236	-0.0539	0.0073	1.10E-13	54.52	-0.0009	0.0051	0.8567	0.0148	0.0208	0.4745	-0.0397	0.0274	0.1480	-0.0080	0.0182	0.6590	-0.0019	0.0161	0.9068	-0.0059	0.0181	0.7454	0.0172	0.0327	0.5992	0.0252	0.0602	0.6752
14	81619945	rs12893151	TSHR	a	c	0.2160	-0.0624	0.0078	1.02E-15	64	-0.0015	0.0053	0.7739	-0.0034	0.0208	0.8693	0.0534	0.0269	0.0468	0.0174	0.0180	0.3356	0.0082	0.0168	0.6267	0.0188	0.0189	0.3213	-0.0439	0.0343	0.2010	-0.0146	0.0630	0.8166
14	93585331	rs8015085	ITPK1	a	g	0.2125	0.0671	0.0077	2.45E-18	75.94	0.0116	0.0055	0.0347	0.0011	0.0209	0.9580	-0.0315	0.0276	0.2536	-0.0211	0.0183	0.2501	0.0158	0.0169	0.3495	0.0164	0.0190	0.3896	-0.0050	0.0347	0.8860	0.0254	0.0634	0.6882
15	49711185	rs17477923	FAM227B/FGF7	t	c	0.7355	0.0826	0.0069	2.57E-33	143.31	-0.0009	0.0049	0.8569	-0.0179	0.0208	0.3891	-0.0165	0.0271	0.5429	0.0086	0.0181	0.6346	0.0077	0.0153	0.6125	0.0143	0.0173	0.4066	0.0006	0.0309	0.9836	-0.0466	0.0584	0.4254
15	49749735	rs11639111	FAM227B/FGF7	t	c	0.4086	0.0450	0.0062	3.60E-13	52.68	0.0060	0.0044	0.1717	-0.0148	0.0208	0.4780	-0.0157	0.0273	0.5656	-0.0022	0.0181	0.9036	-0.0116	0.0136	0.3949	-0.0220	0.0154	0.1521	0.0264	0.0275	0.3360	-0.0206	0.0515	0.6889
15	89113877	rs13329353	DET1	t	c	0.6775	0.0614	0.0065	5.17E-21	89.23	-0.0002	0.0046	0.9641	0.0470	0.0209	0.0246	-0.0079	0.0271	0.7722	0.0260	0.0181	0.1509	-0.0223	0.0143	0.1196	-0.0167	0.0161	0.3016	-0.0302	0.0292	0.3013	-0.1042	0.0545	0.0556
16	4015313	rs1045476	ADCY9	a	g	0.1771	0.0490	0.0082	2.36E-09	35.71	0.0046	0.0057	0.4207	0.0297	0.0217	0.1716	-0.0619	0.0290	0.0327	0.0065	0.0190	0.7316	0.0340	0.0180	0.0590	0.0354	0.0202	0.0807	0.0382	0.0367	0.2980	0.0169	0.0696	0.8085
16	14405428	rs30227	MIR365A	t	c	0.6115	-0.0468	0.0063	7.59E-14	55.18	-0.0062	0.0044	0.1616	0.0124	0.0208	0.5519	-0.0149	0.0271	0.5822	0.0167	0.0181	0.3555	-0.0158	0.0140	0.2577	-0.0086	0.0157	0.5832	-0.0334	0.0285	0.2403	-0.0324	0.0530	0.5415
16	79745487	rs17767491	MAF	a	g	0.6784	0.0883	0.0065	3.35E-42	184.54	-0.0070	0.0046	0.1290	-0.0118	0.0206	0.5681	-0.0325	0.0269	0.2265	-0.0123	0.0180	0.4960	0.0079	0.0143	0.5816	0.0052	0.0161	0.7477	0.0096	0.0289	0.7408	-0.0065	0.0537	0.9037
17	44762589	rs77819282	NSF	a	g	0.2371	0.0452	0.0074	1.13E-09	37.31	NA	NA	NA	NA	NA	NA	NA	NA	NA	NA	NA	NA	NA	NA	NA	NA	NA	NA	NA	NA	NA	NA	NA	NA
17	70121339	rs1042673	SOX9	a	g	0.5214	-0.0546	0.0061	3.57E-19	80.12	-0.0008	0.0043	0.8553	-0.0389	0.0208	0.0619	-0.0074	0.0272	0.7853	-0.0141	0.0181	0.4372	-0.0092	0.0135	0.4959	0.0013	0.0152	0.9346	-0.0433	0.0272	0.1117	-0.0335	0.0515	0.5152
17	70369758	rs963384	SOX9	t	c	0.4644	0.0351	0.0063	2.77E-08	31.04	-0.0041	0.0044	0.3410	0.0275	0.0209	0.1880	-0.0158	0.0273	0.5613	0.0179	0.0182	0.3257	0.0002	0.0138	0.9871	-0.0030	0.0155	0.8487	0.0127	0.0282	0.6531	-0.0254	0.0527	0.6302
19	7222655	rs4804413	INSR	t	c	0.4371	0.0532	0.0062	8.64E-18	73.63	-0.0042	0.0043	0.3347	-0.0183	0.0209	0.3810	-0.0129	0.0273	0.6353	0.0001	0.0181	0.9944	-0.0092	0.0135	0.4927	-0.0158	0.0151	0.2975	0.0068	0.0273	0.8025	0.0185	0.0508	0.7156
20	22596879	rs1203944	FOXA2	t	c	0.2279	-0.0509	0.0073	2.42E-12	48.62	-0.0073	0.0053	0.1637	-0.0065	0.0210	0.7552	-0.0176	0.0275	0.5215	-0.0222	0.0183	0.2262	0.0258	0.0163	0.1138	0.0349	0.0183	0.0570	-0.0158	0.0334	0.6366	-0.0091	0.0609	0.8816
23	3612081	rs12390237	PRKX	a	g	0.6177	-0.0458	0.0068	1.74E-11	45.36	NA	NA	NA	NA	NA	NA	NA	NA	NA	NA	NA	NA	NA	NA	NA	NA	NA	NA	NA	NA	NA	NA	NA	NA

Supplementary Table 4. Variants used as instruments for Mendelian Randomization analyses with normal range FT4 levels as the exposure.

Chr	Pos	RSID	GeneName	Effect_allele	Other_allele	EAF_FT4	Effect_FT4	StdErr_FT4	Pvalue_FT4	Fs	logOR_MDD	StdErr_MDD	Pvalue_MDD	logOR_rMDD	StdErr_rMDD	Pvalue_rMDD	logOR_sMDD	StdErr_sMDD	Pvalue_sMDD	logOR_subMDD	StdErr_subMDD	Pvalue_subMDD	logOR_BD	StdErr_BD	Pvalue_BD	logOR_BD1	StdErr_BD1	Pvalue_BD1	logOR_BD2	StdErr_BD2	Pvalue_BD2	logOR_SABD	StdErr_SABD	Pvalue_SABD
1	54252139	rs145019385	DIO1	t	c	0.9810	0.1813	0.0317	1.13E-08	32.71	0.0042	0.0145	0.7721	NA	NA	NA	NA	NA	NA	NA	NA	NA	-0.0225	0.0548	0.6813	NA	NA	NA	NA	NA	NA	NA	NA	NA
1	54369674	rs12033572	DIO1	c	g	0.0450	0.1152	0.0171	1.45E-11	45.39	-0.0089	0.0106	0.4009	NA	NA	NA	NA	NA	NA	NA	NA	NA	0.0140	0.0357	0.6953	NA	NA	NA	NA	NA	NA	NA	NA	NA
1	54375570	rs2235544	DIO1	a	c	0.5221	0.1387	0.0065	4.20E-101	455.33	-0.0033	0.0043	0.4437	0.0262	0.0208	0.2068	0.0119	0.0272	0.6613	0.0029	0.0181	0.8746	-0.0265	0.0135	0.0489	-0.0305	0.0151	0.0442	0.0052	0.0273	0.8499	-0.0134	0.0513	0.7938
1	54578401	rs954878	DIO1	a	g	0.4464	-0.0582	0.0065	4.83E-19	80.17	0.0009	0.0043	0.8314	-0.0303	0.0208	0.1457	0.0195	0.0272	0.4744	-0.0047	0.0182	0.7960	0.0181	0.0136	0.1829	0.0194	0.0152	0.2032	0.0141	0.0275	0.6075	-0.0414	0.0525	0.4310
2	135632981	rs4954192	ACMSD	t	c	0.4348	-0.0409	0.0071	8.38E-09	33.18	NA	NA	NA	-0.0001	0.0211	0.9955	-0.0283	0.0277	0.3069	0.0021	0.0184	0.9100	0.0139	0.0138	0.3142	0.0217	0.0155	0.1609	-0.0129	0.0282	0.6467	0.0287	0.0527	0.5863
3	181718601	rs6785807	SOX2-OT	a	g	0.1533	-0.0590	0.0093	2.47E-10	40.25	0.0009	0.0058	0.8760	0.0284	0.0205	0.1664	0.0250	0.0269	0.3524	0.0099	0.0179	0.5817	0.0081	0.0186	0.6625	-0.0038	0.0209	0.8560	0.0551	0.0374	0.1406	0.0733	0.0684	0.2839
4	170992760	rs6854291	AADAT	a	g	0.0971	0.1170	0.0114	1.35E-24	105.33	-0.0026	0.0073	0.7199	0.0115	0.0204	0.5724	-0.0540	0.0272	0.0473	0.0095	0.0178	0.5933	-0.0176	0.0231	0.4443	-0.0330	0.0260	0.2030	0.0469	0.0464	0.3123	0.0162	0.0882	0.8545
6	19381386	rs10946313	ID4	t	c	0.6325	0.0455	0.0068	2.28E-11	44.77	-0.0016	0.0045	0.7213	-0.0513	0.0208	0.0136	0.0385	0.0274	0.1605	-0.0297	0.0181	0.1017	0.0285	0.0139	0.0402	0.0417	0.0156	0.0077	-0.0259	0.0278	0.3515	0.1169	0.0536	0.0291
6	25777481	rs9356988	SLC17A4	a	g	0.2725	-0.0510	0.0073	3.56E-12	48.81	0.0031	0.0048	0.5239	-0.0172	0.0209	0.4118	0.0029	0.0273	0.9165	0.0246	0.0182	0.1759	-0.0209	0.0150	0.1648	-0.0176	0.0169	0.2981	-0.0097	0.0303	0.7488	-0.0499	0.0581	0.3905
6	26001742	rs137964359	SLC17A4	t	c	0.9851	-0.1999	0.0315	2.13E-10	40.27	-0.0153	0.0201	0.4489	NA	NA	NA	NA	NA	NA	NA	NA	NA	0.0197	0.0637	0.7574	NA	NA	NA	NA	NA	NA	NA	NA	NA
6	100620931	rs17185536	LOC728012	t	c	0.2387	0.0726	0.0081	1.93E-19	80.33	-0.0197	0.0050	0.0001	-0.0513	0.0207	0.0134	-0.0073	0.0270	0.7877	-0.0505	0.0181	0.0052	-0.0128	0.0162	0.4316	-0.0120	0.0182	0.5104	0.0338	0.0334	0.3108	-0.1374	0.0631	0.0293
8	61212179	rs67583169	CA8	c	g	0.8655	0.0613	0.0095	9.99E-11	41.64	-0.0043	0.0063	0.4901	-0.0043	0.0222	0.8464	0.0589	0.0296	0.0467	-0.0089	0.0194	0.6449	-0.0352	0.0193	0.0673	-0.0347	0.0217	0.1095	-0.0080	0.0394	0.8389	-0.1467	0.0700	0.0361
9	4223660	rs10119187	GLIS3	t	c	0.8138	0.0497	0.0085	4.11E-09	34.19	-0.0028	0.0057	0.6277	0.0137	0.0209	0.5101	0.0369	0.0274	0.1785	0.0410	0.0183	0.0248	-0.0003	0.0175	0.9857	0.0044	0.0197	0.8213	-0.0287	0.0355	0.4185	0.0261	0.0668	0.6960
9	100552559	rs10739496	FOXE1	t	c	0.6637	0.0777	0.0068	4.20E-30	130.56	0.0018	0.0046	0.6997	0.0237	0.0207	0.2541	-0.0464	0.0269	0.0842	-0.0397	0.0180	0.0271	-0.0335	0.0142	0.0181	-0.0389	0.0160	0.0148	-0.0030	0.0289	0.9185	-0.0442	0.0543	0.4157
9	100739117	rs10984606	FOXE1	t	g	0.5018	-0.0398	0.0065	1.17E-09	37.49	0.0037	0.0043	0.3942	0.0238	0.0208	0.2519	0.0304	0.0272	0.2638	0.0120	0.0181	0.5089	0.0375	0.0135	0.0055	0.0368	0.0152	0.0153	0.0461	0.0275	0.0935	0.0715	0.0518	0.1671
9	127015440	rs10818937	NEK6	t	c	0.3144	-0.0475	0.0070	1.31E-11	46.05	0.0135	0.0046	0.0033	0.0400	0.0207	0.0536	0.0357	0.0271	0.1877	0.0209	0.0181	0.2481	0.0043	0.0143	0.7631	-0.0039	0.0161	0.8105	0.0500	0.0287	0.0820	0.0113	0.0547	0.8368
9	139092679	rs4842131	LHX3	t	c	0.4499	-0.1037	0.0075	7.68E-44	191.18	0.0013	0.0045	0.7677	-0.0014	0.0208	0.9469	0.0171	0.0272	0.5298	0.0065	0.0181	0.7214	0.0318	0.0143	0.0262	0.0414	0.0160	0.0095	0.0022	0.0297	0.9415	0.0592	0.0531	0.2650
9	139122363	rs55679545	LHX3	a	g	0.2744	0.0440	0.0076	8.42E-09	33.52	-0.0031	0.0048	0.5181	-0.0286	0.0207	0.1664	-0.0560	0.0272	0.0397	-0.0049	0.0180	0.7840	0.0043	0.0150	0.7766	0.0162	0.0169	0.3373	-0.0210	0.0305	0.4913	-0.0576	0.0571	0.3136
11	47737501	rs11039355	FNBP4	t	c	0.3358	-0.0385	0.0070	3.51E-08	30.25	-0.0198	0.0045	0.0000	NA	NA	NA	NA	NA	NA	NA	NA	NA	-0.0230	0.0143	0.1072	-0.0234	0.0161	0.1462	-0.0199	0.0290	0.4925	0.0927	0.0543	0.0879
12	21331549	rs4149056	SLCO1B1	t	c	0.8430	-0.0506	0.0089	1.34E-08	32.32	-0.0039	0.0060	0.5103	-0.0617	0.0204	0.0025	-0.0466	0.0267	0.0812	-0.0307	0.0179	0.0856	-0.0018	0.0182	0.9230	0.0068	0.0205	0.7424	-0.0531	0.0367	0.1477	-0.0875	0.0680	0.1983
14	80464293	rs150816132	DIO2	a	g	0.0114	-0.2197	0.0398	3.47E-08	30.47	0.0165	0.0210	0.4323	NA	NA	NA	NA	NA	NA	NA	NA	NA	0.0743	0.0790	0.3473	NA	NA	NA	NA	NA	NA	NA	NA	NA
14	80534869	rs978055	DIO2	a	t	0.3779	0.0384	0.0067	1.06E-08	32.85	-0.0047	0.0044	0.2833	0.0191	0.0208	0.3604	0.0071	0.0273	0.7939	-0.0148	0.0182	0.4152	0.0050	0.0138	0.7148	0.0023	0.0155	0.8818	0.0266	0.0280	0.3425	0.0476	0.0513	0.3537
14	80669580	rs225014	DIO2	t	c	0.6403	0.0535	0.0067	1.83E-15	63.76	0.0018	0.0045	0.6821	0.0126	0.0209	0.5462	-0.0005	0.0272	0.9857	0.0028	0.0181	0.8777	0.0103	0.0140	0.4608	0.0104	0.0157	0.5062	0.0110	0.0285	0.7008	0.0123	0.0528	0.8161
14	101852075	rs12323871	DIO3OS	t	c	0.8167	-0.0474	0.0084	1.42E-08	31.84	0.0011	0.0056	0.8449	-0.0448	0.0207	0.0300	0.0156	0.0274	0.5698	-0.0090	0.0181	0.6191	0.0185	0.0172	0.2826	0.0078	0.0193	0.6863	0.0619	0.0357	0.0824	-0.0120	0.0648	0.8526
14	101998443	rs11626434	DIO3OS	c	g	0.3585	0.0583	0.0069	4.08E-17	71.39	0.0032	0.0046	0.4781	0.0100	0.0208	0.6308	0.0090	0.0272	0.7419	0.0431	0.0180	0.0169	-0.0101	0.0145	0.4870	-0.0072	0.0163	0.6579	-0.0173	0.0294	0.5552	0.0357	0.0559	0.5235
15	63873658	rs12907106	USP3	c	g	0.2670	-0.0407	0.0074	3.67E-08	30.25	-0.0028	0.0048	0.5600	-0.0508	0.0213	0.0170	-0.0079	0.0276	0.7755	0.0051	0.0184	0.7802	-0.0178	0.0149	0.2342	-0.0225	0.0168	0.1810	0.0220	0.0301	0.4641	0.0063	0.0558	0.9102
16	12703395	rs8063103	SNX29	c	g	0.8511	-0.0522	0.0092	1.61E-08	32.19	0.0036	0.0061	0.5536	0.0039	0.0210	0.8511	0.0093	0.0276	0.7363	0.0305	0.0184	0.0983	-0.0077	0.0193	0.6918	-0.0064	0.0217	0.7672	0.0101	0.0401	0.8014	-0.1008	0.0705	0.1525
17	16049626	rs11078333	NCOR1	a	t	0.5307	0.0513	0.0072	9.91E-13	50.77	-0.0036	0.0043	0.4013	-0.0595	0.0209	0.0044	-0.0304	0.0273	0.2651	-0.0229	0.0182	0.2083	0.0027	0.0143	0.8486	-0.0004	0.0161	0.9818	-0.0243	0.0290	0.4027	0.0968	0.0549	0.0780
18	29173795	rs1080094	SLC25A52	a	g	0.6030	-0.0415	0.0066	4.06E-10	39.54	-0.0108	0.0044	0.0143	-0.0018	0.0210	0.9329	0.0036	0.0275	0.8957	0.0109	0.0184	0.5513	-0.0059	0.0137	0.6685	-0.0183	0.0154	0.2347	0.0256	0.0281	0.3616	0.0110	0.0518	0.8320
18	29306737	rs113107469	SLC25A52	t	c	0.0294	0.1996	0.0220	1.00E-19	82.31	0.0235	0.0125	0.0605	NA	NA	NA	NA	NA	NA	NA	NA	NA	0.0200	0.0443	0.6508	NA	NA	NA	NA	NA	NA	NA	NA	NA
18	57914644	rs56069042	MC4R	a	g	0.9600	0.1061	0.0186	1.16E-08	32.54	0.0169	0.0120	0.1592	NA	NA	NA	NA	NA	NA	NA	NA	NA	0.0252	0.0393	0.5212	NA	NA	NA	NA	NA	NA	NA	NA	NA

Supplementary Table 5. Power calculations using a non-centrality parameter-based approach, implemented in a publicly available mRnd web tool (http://cnsgenomics.com/shiny/mRnd/).

exposure	outcome	sample_size	α	actual_OR_{IVW}	P_{IVW}	*K*	R^2	detectable_OR_{IVW}
TSH	MDD	500199	0.05	1.0049	0.6762	0.3414	0.094	<0.9731 / >1.0274
TSH	rMDD	80933	0.05	0.9986	0.9780	0.2156	0.094	<0.9237 / >1.0789
TSH	sMDD	75506	0.05	0.9162	0.1558	0.1592	0.094	<0.9110 / >1.0916
TSH	subMDD	85078	0.05	1.0629	0.1340	0.2538	0.094	<0.9297 / >1.0729
TSH	BD	51710	0.05	0.9661	0.3127	0.3936	0.094	<0.9208 / >1.0844
TSH	BD1	45871	0.05	0.9972	0.9468	0.3244	0.094	<0.9121 / >1.0933
TSH	BD2	25576	0.05	0.9179	0.2235	0.1338	0.094	<0.8371 / >1.1704
TSH	SABD	9667	0.05	0.8168	0.1258	0.1011	0.094	<0.7040 / >1.3148
FT4	MDD	500199	0.05	0.9877	0.5560	0.3414	0.048	<0.9626 / >1.0384
FT4	rMDD	80933	0.05	1.0440	0.6770	0.2156	0.048	<0.8939 / >1.1112
FT4	sMDD	75506	0.05	0.9012	0.2862	0.1592	0.048	<0.8762 / >1.1289
FT4	subMDD	85078	0.05	0.9214	0.2701	0.2538	0.048	<0.9024 / >1.1027
FT4	BD	51710	0.05	0.8872	0.0223	0.3936	0.048	<0.8907 / >1.1196
FT4	BD1	45871	0.05	0.8662	0.0469	0.3244	0.048	<0.8784 / >1.1320
FT4	BD2	25576	0.05	0.9186	0.3910	0.1338	0.048	<0.7743 / >1.2402
FT4	SABD	9667	0.05	0.8825	0.5587	0.1011	0.048	<0.5923 / >1.4443

Abbreviation:	Explanation:
**sample_size**	sample size of the underlying GWAS on the outcome
**α**	Type-I error rate
**actual_OR_{IVW}**	OR from the MR analysis with the IVW method
**P_{IVW}**	P-value from the MR analysis with the IVW method
K	Proportion of cases in the GWAS on the outcome
**R^2**	Proportion of variance in the exposure explained by the genetic variants used as instruments
**detectable_OR_{IVW}**	Smallest detectable odds ratio (power = 0.8, α = 0.05) in the MR analysis

Supplementary Table 6. Results of the Mendelian Randomization (MR) analyses investigating the association between genetically predicted TSH and FT4 levels and the tested outcomes using various MR methods.

Outcome	Method	Exposure: TSH levels					Exposure: FT4 levels				
		$N_{instruments}$	OR	LB	UB	*P*	$N_{instruments}$	OR	LB	UB	*P*
MDD	IVW	55	1.0049	0.9817	1.0286	0.6762	30	0.9877	0.9466	1.0306	0.5560
	MR Egger	55	0.9801	0.9248	1.0387	0.4906	30	0.9875	0.8960	1.0883	0.7932
	WM	55	0.9941	0.9621	1.0272	0.7177	30	0.9774	0.9373	1.0192	0.2738
	MR PRESSO	55	1.0049	0.9817	1.0286	0.6762	28	0.9910	0.9611	1.0219	0.5516
rMDD	IVW	48	0.9986	0.9026	1.1049	0.9780	24	1.0440	0.8452	1.2896	0.6770
	MR Egger	48	1.0632	0.8031	1.4076	0.6622	24	1.1667	0.6674	2.0397	0.5728
	WM	48	0.9560	0.8302	1.1009	0.5241	24	1.1434	0.9294	1.4067	0.1940
	MR PRESSO	48	0.9986	0.9026	1.1049	0.9780	23	1.0776	0.8813	1.3177	0.4489
sMDD	IVW	48	0.9162	0.8109	1.0351	0.1558	24	0.9012	0.7400	1.0975	0.2862
	MR Egger	48	1.0500	0.7500	1.4699	0.7718	24	0.8018	0.4761	1.3503	0.3889
	WM	48	0.8753	0.7293	1.0506	0.1490	24	0.8831	0.6740	1.1570	0.3510
	MR PRESSO	48	0.9162	0.8109	1.0351	0.1558	24	0.9012	0.7400	1.0975	0.2862
subMDD	IVW	48	1.0629	0.9807	1.1519	0.1340	24	0.9214	0.7930	1.0704	0.2701
	MR Egger	48	1.0245	0.8210	1.2784	0.8271	24	1.0379	0.6997	1.5396	0.8466
	WM	48	1.1124	0.9926	1.2466	0.0662	24	0.9533	0.7934	1.1455	0.5955
	MR PRESSO	48	1.0629	0.9950	1.1354	0.0695	23	0.8953	0.7781	1.0301	0.1163
BD	IVW	55	0.9661	0.9027	1.0339	0.3127	31	0.8872	0.8016	0.9819	**0.0223**
	MR Egger	55	0.8915	0.7527	1.0559	0.1792	31	0.7610	0.6065	0.9548	**0.0200**
	WM	55	0.9940	0.9012	1.0963	0.9019	31	0.8369	0.7255	0.9654	**0.0163**
	MR PRESSO	55	0.9661	0.9027	1.0339	0.3127	31	0.8872	0.8016	0.9819	**0.0223**
BD1	IVW	48	0.9972	0.9161	1.0855	0.9468	25	0.8662	0.7519	0.9979	**0.0469**
	MR Egger	48	0.9375	0.7461	1.1781	0.5725	25	0.5954	0.4327	0.8194	**0.0027**
	WM	48	1.0225	0.9086	1.1508	0.7063	25	0.8107	0.6828	0.9626	**0.0187**
	MR PRESSO	48	0.9972	0.9161	1.0855	0.9468	24	0.8435	0.7429	0.9577	**0.0109**
BD2	IVW	48	0.9179	0.7981	1.0556	0.2235	25	0.9186	0.7516	1.1227	0.3910
	MR Egger	48	0.6553	0.4511	0.9521	**0.0274**	25	1.3191	0.7950	2.1886	0.2696
	WM	48	1.0101	0.8124	1.2557	0.9267	25	0.9915	0.7450	1.3195	0.9514
	MR PRESSO	48	0.9179	0.7985	1.0551	0.2221	25	0.9186	0.7612	1.1085	0.3604
SABD	IVW	48	0.8168	0.6290	1.0606	0.1258	25	0.8825	0.5711	1.3636	0.5587
	MR Egger	48	0.6731	0.3351	1.3522	0.2593	25	0.6564	0.2156	1.9987	0.4421
	WM	48	0.7552	0.5094	1.1196	0.1580	25	0.8899	0.5060	1.5651	0.6737
	MR PRESSO	48	0.8168	0.6694	0.9966	**0.0464**	25	0.8825	0.5711	1.3636	0.5587

Supplementary Table 7. Intercept values from the Mendelian Randomization analyses investigating the association between genetically predicted TSH and FT4 levels and the tested outcomes using the MR Egger method.

Outcome	Exposure: TSH levels					Exposure: FT4 levels				
	**intercept**	**StdErr**	**LB**	**UB**	***P*** $_{intercept}$	**intercept**	**StdErr**	**LB**	**UB**	***P*** $_{intercept}$
**MDD**	0.0018	0.0019	-0.0020	0.0056	0.3501	1.4E-05	0.0033	-0.0067	0.0068	0.9966
rMDD	-0.0044	0.0092	-0.0228	0.0140	0.6316	-0.0079	0.0176	-0.0443	0.0286	0.6592
sMDD	-0.0096	0.0110	-0.0317	0.0125	0.3859	0.0083	0.0164	-0.0257	0.0422	0.6192
subMDD	0.0026	0.0072	-0.0119	0.0171	0.7211	-0.0084	0.0124	-0.0341	0.0173	0.5041
**BD**	0.0058	0.0056	-0.0054	0.0169	0.3034	0.0115	0.0075	-0.0038	0.0267	0.1346
BD1	0.0042	0.0072	-0.0103	0.0187	0.5606	0.0259	0.0098	0.0057	0.0462	**0.0144**
BD2	0.0231	0.0118	-0.0006	0.0468	0.0563	-0.0250	0.0155	-0.0571	0.0071	0.1209
SABD	0.0132	0.0220	-0.0310	0.0575	0.5501	0.0205	0.0342	-0.0502	0.0911	0.5550

Supplementary Table 8. Detailed results of the Mendelian Randomization (MR) analyses investigating the association between genetically predicted TSH and FT4 levels and the tested outcomes using the MR PRESSO method.

A. Exposure: TSH levels

Outcome	Global test	Outliers test					Distortion test
	P_glob	Outliers	OR_corr	LB_corr	UB_corr	P_corr	P_dist
**MDD**	0.115	no outliers	NA	NA	NA	NA	NA
rMDD	0.149	no outliers	NA	NA	NA	NA	NA
sMDD	0.451	no outliers	NA	NA	NA	NA	NA
subMDD	0.964	no outliers	NA	NA	NA	NA	NA
**BD**	0.333	no outliers	NA	NA	NA	NA	NA
BD1	0.167	no outliers	NA	NA	NA	NA	NA
BD2	0.493	no outliers	NA	NA	NA	NA	NA
SABD	0.993	no outliers	NA	NA	NA	NA	NA

B. Exposure: FT4 levels

Outcome	Global test	Outliers test					Distortion test
	P_glob	Outliers	OR_corr	LB_corr	UB_corr	P_corr	P_dist
**MDD**	<0,001	rs11039355, rs17185536	0.9910	0.9611	1.0219	0.5516	0.654
rMDD	<0,001	rs11078333	1.0776	0.8813	1.3177	0.4489	0.820
sMDD	0.218	no outliers	NA	NA	NA	NA	NA
subMDD	0.028	rs11626434	0.8953	0.7781	1.0301	0.1163	0.757
**BD**	0.191	no outliers	NA	NA	NA	NA	NA
BD1	0.032	rs10946313	0.8435	0.7429	0.9577	**0.0109**	0.696
BD2	0.660	no outliers	NA	NA	NA	NA	NA
SABD	0.112	no outliers	NA	NA	NA	NA	NA

Supplementary Table 9. Results of the Mendelian Randomization (MR) analyses using as instruments two specific subsets of TSH associated variants, including: (i) variants associated with autoimmune thyroid disease ("AITD vairants"), and (ii) other genetic variants associated with TSH levels ("non-AITD variants") in the GWAS by Teumer et al.

Outcome	Analysis type	Instruments	Exposure: TSH levels				
			$N_{instruments}$	OR	LB	UB	*P*
MDD	main	all available	55	1.0049	0.9817	1.0286	0.6762
	sensitivity	AITD variants	14	0.9806	0.9269	1.0373	0.4645
	sensitivity	non-AITD variants	41	1.0132	0.9873	1.0397	0.3122
rMDD	main	all available	48	0.9986	0.9026	1.1049	0.9780
	sensitivity	AITD variants	14	0.9215	0.7521	1.1291	0.4006
	sensitivity	non-AITD variants	34	1.0369	0.9191	1.1699	0.5450
sMDD	main	all available	48	0.9162	0.8109	1.0351	0.1558
	sensitivity	AITD variants	14	0.9513	0.7240	1.2499	0.6992
	sensitivity	non-AITD variants	34	0.9001	0.7762	1.0438	0.1577
subMDD	main	all available	48	1.0629	0.9807	1.1519	0.1340
	sensitivity	AITD variants	14	1.0816	0.9281	1.2605	0.2881
	sensitivity	non-AITD variants	34	1.0542	0.9553	1.1634	0.2837
BD	main	all available	55	0.9661	0.9027	1.0339	0.3127
	sensitivity	AITD variants	14	0.9912	0.8184	1.2004	0.9219
	sensitivity	non-AITD variants	41	0.9577	0.8875	1.0335	0.2588
BD1	main	all available	48	0.9972	0.9161	1.0855	0.9468
	sensitivity	AITD variants	13	1.0465	0.8341	1.3129	0.6702
	sensitivity	non-AITD variants	35	0.9797	0.8939	1.0737	0.6519
BD2	main	all available	48	0.9179	0.7981	1.0556	0.2235
	sensitivity	AITD variants	13	0.8084	0.6044	1.0814	0.1372
	sensitivity	non-AITD variants	35	0.9622	0.8094	1.1438	0.6534
SABD	main	all available	48	0.8168	0.6290	1.0606	0.1258
	sensitivity	AITD variants	13	0.6736	0.3906	1.1618	0.1402
	sensitivity	non-AITD variants	35	0.8769	0.6440	1.1942	0.3934

Supplementary Table 10. Results of the Mendelian Randomization (MR) analyses using as instruments two specific subsets of FT4 associated variants, including: (i) variants within the deiodinases loci ("DIO vairants"), and (ii) other genetic variants associated with FT4 levels ("non-DIO variants") in the GWAS by Teumer et al.

Outcome	Analysis type	Instruments	Exposure: FT4 levels				
			$N_{instruments}$	OR	LB	UB	*P*
MDD	main	all available	30	0.9877	0.9466	1.0306	0.5560
	sensitivity	DIO variants	7	0.9752	0.9209	1.0327	0.3241
	sensitivity	non-DIO variants	23	0.9948	0.9364	1.0569	0.8610
rMDD	main	all available	24	1.0440	0.8452	1.2896	0.6770
	sensitivity	DIO variants	4	1.2871	0.8600	1.9264	0.1404
	sensitivity	non-DIO variants	20	0.9680	0.7444	1.2589	0.7986
sMDD	main	all available	24	0.9012	0.7400	1.0975	0.2862
	sensitivity	DIO variants	4	1.0289	0.6072	1.7435	0.8745
	sensitivity	non-DIO variants	20	0.8588	0.6696	1.1014	0.2158
subMDD	main	all available	24	0.9214	0.7930	1.0704	0.2701
	sensitivity	DIO variants	4	1.0094	0.7103	1.4346	0.9375
	sensitivity	non-DIO variants	20	0.8915	0.7371	1.0783	0.2217
BD	main	all available	31	0.8872	0.8016	0.9819	**0.0223**
	sensitivity	DIO variants	7	0.8683	0.7218	1.0446	0.1108
	sensitivity	non-DIO variants	24	0.8974	0.7825	1.0292	0.1159
BD1	main	all available	25	0.8662	0.7519	0.9979	**0.0469**
	sensitivity	DIO variants	4	0.8367	0.6233	1.1233	0.1497
	sensitivity	non-DIO variants	21	0.8816	0.7322	1.0614	0.1719
BD2	main	all available	25	0.9186	0.7516	1.1227	0.3910
	sensitivity	DIO variants	4	1.0549	0.6193	1.7971	0.7702
	sensitivity	non-DIO variants	21	0.8561	0.6673	1.0983	0.2080
SABD	main	all available	25	0.8825	0.5711	1.3636	0.5587
	sensitivity	DIO variants	4	1.1146	0.4100	3.0301	0.7527
	sensitivity	non-DIO variants	21	0.7855	0.4449	1.3868	0.3861

Supplementary Table 11. Results of the Mendelian Randomization analyses investigating the effects of MDD and BD on TSH and FT4 levels.

Outcome	Method	Exposure: MDD					Exposure: BD				
		$N_{instruments}$	β	LB	UB	*P*	$N_{instruments}$	β	LB	UB	*P*
**TSH**	IVW	102	-0.0450	-0.1015	0.0116	0.1180	22	-0.0304	-0.0756	0.0148	0.1759
	MR Egger	102	-0.2627	-0.6012	0.0757	0.1267	22	-0.1342	-0.4029	0.1346	0.3101
	WM	102	-0.0528	-0.1293	0.0237	0.1738	22	-0.0109	-0.0722	0.0504	0.7151
	MR PRESSO	102	-0.0450	-0.1015	0.0116	0.1180	22	-0.0304	-0.0756	0.0148	0.1759
**FT4**	IVW	102	-0.0223	-0.0849	0.0402	0.4802	22	0.0062	-0.0390	0.0514	0.7798
	MR Egger	102	-0.2111	-0.5834	0.1613	0.2635	22	0.1330	-0.1371	0.4031	0.3165
	WM	102	-0.0029	-0.0899	0.0841	0.9479	22	0.0189	-0.0436	0.0814	0.5368
	MR PRESSO	102	-0.0223	-0.0849	0.0402	0.4802	22	0.0062	-0.0390	0.0514	0.7798
